# The lipopeptide Pam_3_CSK_4_ inhibits Rift Valley fever virus infection and protects from encephalitis

**DOI:** 10.1371/journal.ppat.1012343

**Published:** 2024-06-27

**Authors:** Trevor Griesman, Cynthia M. McMillen, Seble Getenet Negatu, Jesse J. Hulahan, Kanupriya Whig, Lenka Dohnalová, Mark Dittmar, Christoph A. Thaiss, Kellie A. Jurado, David C. Schultz, Amy L. Hartman, Sara Cherry

**Affiliations:** 1 Department of Pathology and Laboratory Medicine, University of Pennsylvania, Philadelphia Pennsylvania, United States of America; 2 Center for Vaccine Research, University of Pittsburgh, Pittsburgh, Pennsylvania, United States of America; 3 Department of Infectious Diseases and Microbiology, University of Pittsburgh School of Public Health, Pittsburgh, Pennsylvania, United States of America; 4 Department of Microbiology, University of Pennsylvania, Philadelphia Pennsylvania, Unites States of America; 5 High throughput screening core, University of Pennsylvania, Philadelphia, Pennsylvania, United States of America; National Institute of Allergy and Infectious Diseases, UNITED STATES

## Abstract

Rift Valley fever virus (RVFV) is an encephalitic bunyavirus that can infect neurons in the brain. There are no approved therapeutics that can protect from RVFV encephalitis. Innate immunity, the first line of defense against infection, canonically antagonizes viruses through interferon signaling. We found that interferons did not efficiently protect primary cortical neurons from RVFV, unlike other cell types. To identify alternative neuronal antiviral pathways, we screened innate immune ligands and discovered that the TLR2 ligand Pam_3_CSK_4_ inhibited RVFV infection, and other bunyaviruses. Mechanistically, we found that Pam_3_CSK_4_ blocks viral fusion, independent of TLR2. In a mouse model of RVFV encephalitis, Pam_3_CSK_4_ treatment protected animals from infection and mortality. Overall, Pam_3_CSK_4_ is a bunyavirus fusion inhibitor active in primary neurons and the brain, representing a new approach toward the development of treatments for encephalitic bunyavirus infections.

## Author summary

Rift Valley fever virus (RVFV) is an emerging mosquito-borne pathogen, which has shown the potential to spread geographically, and which can cause severe disease in humans. One of the most serious complications of RVFV infection is invasion into the brain. There are no approved treatments for RVFV infection, or any other bunyavirus that infects the brain. We found that the classically antiviral interferon pathway was not protective in neurons. This led us to screen for new antivirals, and we identified Pam_3_CSK_4_, which inhibited RVFV and related bunyaviruses, but not vesicular stomatitis virus. We found that the antiviral activity of Pam_3_CSK_4_ was separate from its previously characterized activity as a TLR2 agonist. Instead, Pam_3_CSK_4_ blocked RVFV entry at the step of viral membrane fusion. Importantly, we found that Pam_3_CSK_4_ prevented RVFV infection in the mouse brain. This work reveals that Pam_3_CSK_4_ prevents encephalitic bunyavirus infection in neurons, a vulnerable cell type, and future research should evaluate the therapeutic potential of Pam_3_CSK_4_.

## Introduction

Rift Valley fever virus (*Phlebovirus riftense*, RVFV) is an emerging, arthropod-borne *Phenuivirus* that can cause encephalitis in humans. RVFV is endemic in sub-Saharan Africa and has spread since its discovery, causing outbreaks across the continent, the surrounding islands, and the Arabian Peninsula [1–3]. The World Health Organization has designated RVFV as a research priority because of its broad vector tropism and potential to severely impact humans and agriculturally important animals [4]. In ruminant animals, RVFV causes abortions and is highly pathogenic in newborns [5,6]. Humans can become infected through contact with infected fluids, or by mosquito bite. Many human infections are asymptomatic or produce flu-like illness, but 8–10% develop severe symptoms including eye disease, hemorrhage, and meningoencephalitis [6–9]. Neurologic disease is characterized by symptoms such as disorientation, hallucinations, vertigo, and/or coma, which often present days or weeks after the onset of infection [7–9]. There are no approved treatments for RVFV infection in humans, and few antivirals in development that target the neurologic stage of disease, although the delayed appearance of neurologic symptoms may provide a temporal window for the delivery of antivirals.

Antiviral therapeutics often target viral entry, such as neutralizing antibodies and fusion inhibitors that have been used to treat human immunodeficiency virus (HIV) and severe acute respiratory syndrome-coronavirus-2[10–12]. RVFV entry may also be a promising target for antiviral inhibition. RVFV enters host cells using the glycoproteins Gn and Gc [13,14]. RVFV Gn on the virion surface can bind the host protein low-density lipoprotein receptor-related protein 1 (LRP1) [15]. Next, RVFV is endocytosed, and membrane fusion is initiated when the endosomal pH drops below ~5.5, triggering the rearrangement of RVFV Gc, a class II fusion protein [13,14]. Treatments which block RVFV fusion can inhibit infection *in vitro*, including endosomal acidification inhibitors [13,16,17], Gc binding peptides [18], virion membrane-intercalating amphipathic compounds [19], and fusion-inhibiting antibodies [20]. However, it is unknown whether such antivirals can decrease RVFV infection in neurons or ameliorate encephalitic disease.

Neurons, a primary target of RVFV infection in the brain [21], are essential, terminally differentiated, long-lived, and largely non-replenished cells. The brain is protected by the blood-brain barrier, which restricts the ingress of cells and pathogens from the blood, yet RVFV can surmount this barrier. In neurons, as in all cells, the innate immune system detects and responds to infections. Innate immunity is characterized by two canonical pathways: the inflammatory nuclear factor kappa-light-chain-enhancer of activated B cells (NF-κB) pathway and antiviral interferon (IFN) pathway. RVFV infection of the brain induces NF-κB and type-I IFN responses [7,21], but it is unclear if innate immunity is protective in this tissue, as inflammation in the brain can lead to encephalitic death [22]. It is hypothesized that innate immune responses in neurons are regulated or executed differently to avoid damage [23,24]. Indeed, while IFN pretreatment is broadly antiviral, IFN-exposed neurons remain vulnerable to viral infections [25–28]. Although little is known about neuronal immunity during RVFV infection, the distantly related *Peribunyavirus*, La Crosse virus (LACV, *Orthobunyavirus lacrosseense*) readily infects neurons in the mouse brain, even while neighboring astrocytes and microglia produce IFNβ [29–31]. Further, IFNβ only partially protects neuronal organoids from LACV [32]. It is unknown whether type-I IFN can prevent RVFV infection in primary neurons.

We set out to identify immune stimuli that inhibited neuronal RVFV infection. We found that treating neurons with IFNs led to the transcription of interferon stimulated genes (ISGs) including antiviral effectors which directly antagonize viral processes like interferon induced protein with tetratricopeptide repeats 1–3 (IFIT1-3) [33], as well as chemokines such as CXC-motif ligand 10 (CXCL10, also known as IP-10)[22]. However, IFN pretreatment weakly blocked RVFV replication in neurons compared to non-neuronal cells. These data suggest that IFN signaling is not sufficient to control RVFV infection in neurons.

To identify alternative immune agonists with anti-RVFV activity in neurons, we screened a diverse library of innate immune ligands. We identified two Toll-like receptor 2 (TLR2) agonists that were protective against RVFV infection. We found that the synthetic lipopeptide Pam_3_CSK_4_ blocked infection by RVFV and LACV in neurons and non-neuronal cells. TLR2 inflammatory signaling was not required for Pam_3_CSK_4_ antiviral activity. Rather, we found that Pam_3_CSK_4_ inhibited RVFV fusion. Pam_3_CSK_4_ was active *in vivo*, as treatment prevented RVFV infection in the mouse brain and protected from mortality. These findings reveal a novel function of Pam_3_CSK_4_, a molecule that has potential in the control of encephalitic bunyavirus infection.

## Results

### Interferons do not efficiently protect neurons from RVFV infection

**Fig 1:**
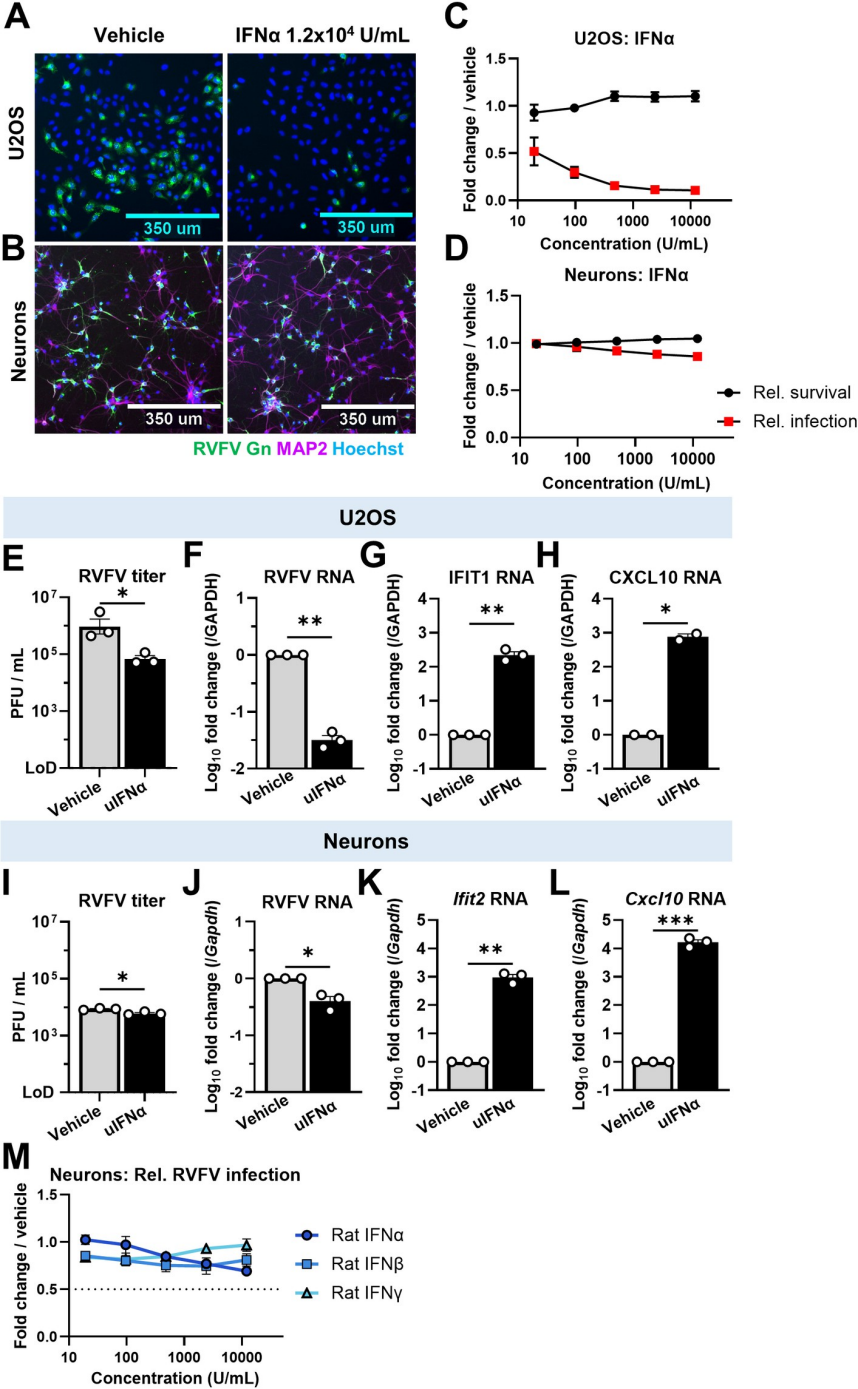
Type I IFNs do not potently protect neurons from RVFV infection. **(A-D**) Human U2OS cells (A,C), or rat cortical neurons (B,D) were treated with vehicle or universal IFNα for 4h before infection with RVFV (24hpi, U2OS: MOI 1, neurons: MOI 0.3). Immunofluorescence and automated microscopy were used to detect RVFV Gn (green) or neuronal MAP2 (magenta). A and B show representative images of cells treated with vehicle or 1.2 x 10^4^ Units / mL at 10x magnification, scale bars = 350μm. C and D show quantification of relative infection (red squares) and survival (black circles) in U2OS (C) or neurons (D). Automated image analysis was used to determine the percentage RVFV Gn positive U2OS cells, or the percentage of neurons (MAP2 positive cells) positive for RVFV Gn. Infection and survival normalized to % infected or total cell number in vehicle treated, infected cells. IC50 values were determined by nonlinear regression. n = 3. **(E,F**) U2OS were treated with vehicle or 1.2 x 10^4^ U/mL uIFNα for 4h before RVFV infection (MOI 1). At 15hpi viral titers in the supernatant were measured by plaque assay (E) or at 24hpi viral RNA was quantified by qPCR (F). LoD = limit of detection. *P = .0167, **P = .0028 **(G-H)** Relative RNA expression of interferon stimulated genes in U2OS treated with 1.2 x 10^4^ U/mL of uIFNα for 4h was measured by qPCR. *P = .0202, **P = .0017 **(I-J)** Neurons were treated and analyzed as in E and F, but infected at an MOI of 0.1. I: *P = .0137, J: *P = .041 **(K-L)** Neurons were treated and analyzed as in G and H. **P = .0013, ***P = .0005 **(M)** Quantification of relative RVFV infection in neurons treated with rat IFN alpha (dark blue circles), rat IFN beta (teal squares), or rat IFN gamma (light blue triangles). Images acquired as in (D), n = 3. Dotted line represents 50% reduction relative to control. For all qPCR experiments, expression was calculated relative to vehicle, normalized to GAPDH (human) or *Gapdh* (Rat) as a loading control. Symbols and bars represent mean, error bars = SEM. Statistical analyses were performed using student’s t test on log_10_ transformed titers (E,I) or Welch’s t test (F-H,J-L).

Previous studies of RVFV have shown that type I interferon (IFN) responses are important in controlling infection peripherally [34–36]. However, it has not been tested if IFNs are protective in the brain, as IFN-treated neurons remain susceptible to some viral infections [25,26,28,30,37]. We tested this *in vitro* using the biosafety level two strain RVFV MP-12, which is attenuated by nine amino acid substitutions across all three genomic segments [38]. At baseline, RVFV MP-12 readily infected primary rat (*Rattus norvegicus*) cortical neurons and human-derived U2OS osteosarcoma cells, a non-neuronal cell (S1A and S1B Fig). We pretreated neurons or U2OS cells for four hours with several doses of universal IFNα [39] or vehicle (water) before infecting with RVFV, and quantified infection by immunofluorescence microscopy 24 hours post infection (hpi; Fig 1A–1D). A high dose of 12,000 Units/mL of IFNα blocked infection of U2OS, but had little effect in neurons, suggesting that IFN treatment of neurons is not protective against RVFV (Fig 1A–1D). In U2OS, the inhibitory concentration 50% (IC50) of universal IFNα was 16.9 U/mL, while in neurons 12,000 U/mL of IFNα failed to reduce infection to 50% of control (Fig 1C and 1D). We observed no IFN-induced toxicity in either cell type (Fig 1C and 1D).

We complemented these microscopy-based studies with plaque assays and real-time, reverse transcription quantitative polymerase chain reaction (qPCR) to measure viral RNA. In U2OS cells, a four hour pretreatment with 12,000 U/mL of universal IFNα reduced RVFV titers from 9.3 x 10^5^ to 6.8 x 10^4^ plaque forming units (PFU) / mL at 16 hpi, and decreased RVFV RNA at 24 hpi by ~30 fold (Fig 1E and 1F). In neurons, the same treatment reduced RVFV titers from 8.7 x 10^3^ to 6.1 x 10^3^ PFU / mL, and decreased RVFV RNA ~2-fold compared to vehicle (Fig 1I and 1J). While these decreases were statistically significant, the small magnitude of change in neurons suggests that IFN treatment is not as potent in neurons as in other cell types.

To evaluate whether neurons and U2OS respond to IFN, we stimulated with 12,000 U/mL of universal IFNα or vehicle for four hours and measured ISG transcription by qPCR. We measured IFIT1 in U2OS cells as it is well known to be transcribed and translated in these cells following IFN stimulation [40]. In neurons, we measured *Ifit2* because it has been reported that *Ifit1* basal expression can be elevated in some subpopulations of mouse neurons [28]. In both U2OS and rat neurons, we observed multi-log increases in the RNA levels of ISGs (Fig 1G, 1H, 1K and 1L). We conclude that both cell types detect and respond to IFNα.

To test whether species-specific IFNs would be more active than universal IFNα, we pretreated rat cortical neurons with rat IFNα, IFNβ, or IFNγ and evaluated RVFV infection by microscopy and qPCR. None of the rat IFNs tested decreased infection below 50% of control levels at 12,000 U/mL (Fig 1M), or significantly reduced RVFV RNA (S1C Fig). These data support our finding that IFN responses are induced but insufficient to control RVFV infection in neurons. Therefore, we set out to identify alternative innate immune ligands that could block neuronal RVFV infection.

### Pam_3_CSK_4_ and LPS-*Rhodobacter sphaeroides* protect against RVFV infection

**Fig 2:**
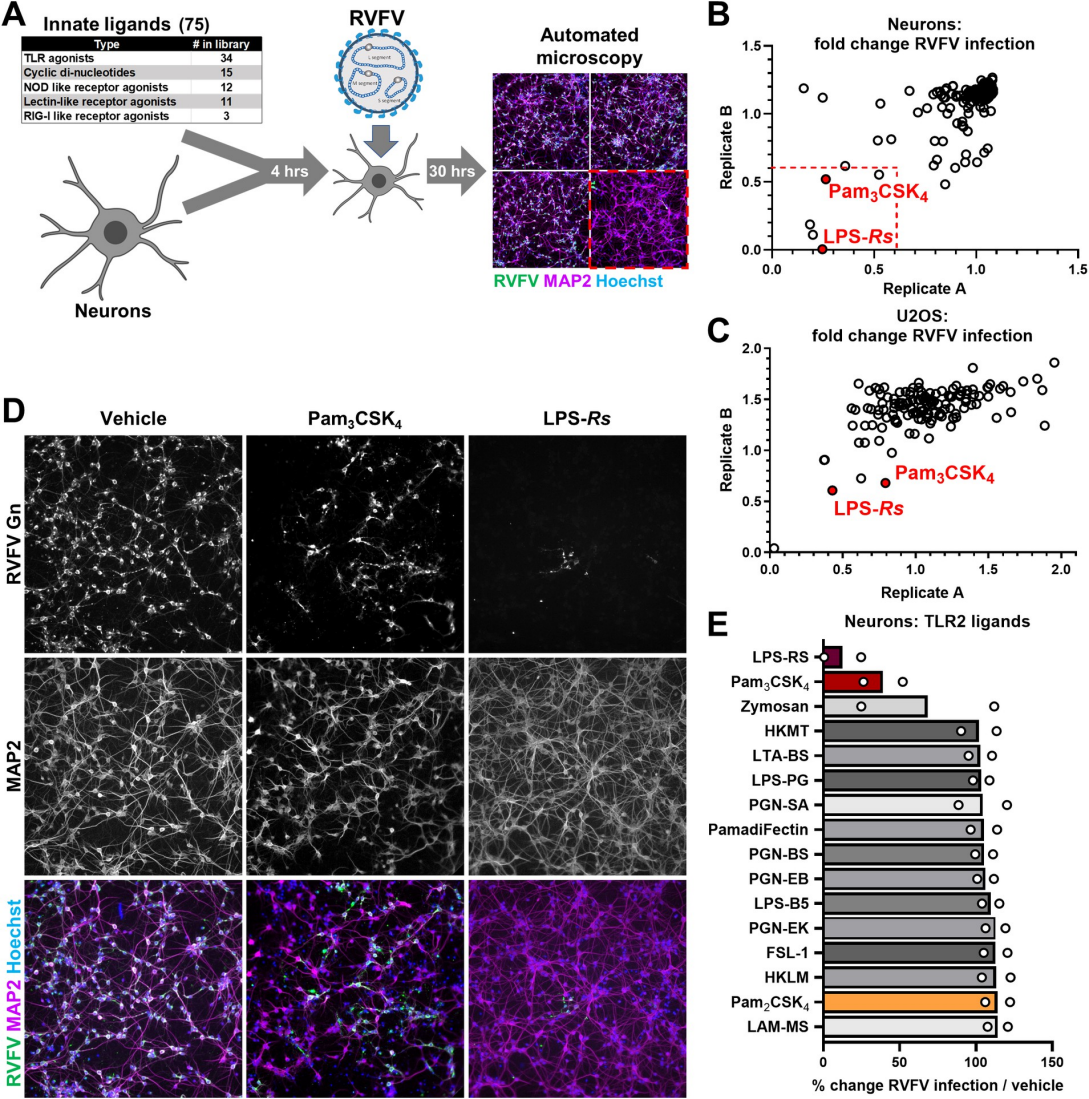
Pam_3_CSK_4_ and LPS-*Rs* protect primary neurons from RVFV infection. **(A)** Diagram of screening workflow. **(B,C)** Results from microscopy-based screening of innate ligands in neurons (B) or U2OS (C) infected with RVFV (neuron MOI 0.1, U2OS MOI 0.35), as detailed in (A). Four images were captured per well and averaged. Each dot represents a ligand. Plots show infection relative to vehicle from duplicate screens. Dotted red lines indicate 60% of vehicle infection levels. Antiviral TLR2 ligands are labelled. **(D)** Representative images from neuron PAMP screens, showing vehicle (water), Pam_3_CSK_4_ (10μg/mL), or LPS-*Rs* (100μg/mL) treated cells at 10x magnification. Neurons are MAP2 positive (magenta), while infected cells are positive for RVFV Gn (green). **(E)** A subset of screen data, showing relative RVFV infection in neurons treated with TLR2 ligands. Bars show mean of two replicates. LPS-*Rs* and Pam_3_CSK_4_ are colored red, while Pam_2_CSK_4_ is shown in orange.

We assembled a diverse panel of 75 immune ligands [41] and tested their antiviral activity against RVFV using a microscopy-based screening workflow, shown in Fig 2A, where we quantified nuclei, neurons (MAP2 positive cells), and infection (RVFV positive cells). Drug treatment was generally well tolerated, though three TLR7 ligands (Gardiquimod, Imiquimod, and Adilipoline) and a Dectin 2 ligand (Furfurman) were neurotoxic (S2A and S2B Fig). Five ligands reduced infection to below 60% of vehicle without impacting neuronal survival: Pam_3_CSK_4_, Lipopolysaccharide-*Rhodobacter sphaeroides (*LPS*-Rs)*, β-glucan peptide, scleroglucan, and Poly(A:U) (Fig 2B). Both Pam_3_CSK_4_ and LPS-*Rs* are Toll-like receptor 2 (TLR2) ligands. TLR2 forms heterodimers with either TLR1 or TLR6 at the plasma membrane to recognize diverse microbial ligands including di- and tri-acylated bacterial lipoproteins, viral glycoproteins, and endogenous ligands [42–46]. TLR2 activation promotes NF-κB signaling, driving the transcription of genes such as tumor necrosis factor alpha (TNFα), CXC-motif ligand 1 (CXCL1, also known as GRO or KC), and CXCL10[47]. Pam_3_CSK_4_, a synthetic, tri-acylated lipopeptide, mimics bacterial lipoprotein anchors and activates TLR2/1 heterodimers (structure in S3 Fig)[43,48]. LPS-*Rs* is known to antagonize TLR4 and contains contaminating lipoproteins which can activate TLR2. Treatment with Pam_3_CSK_4_ or LPS-*Rs* decreased infection in neurons relative to vehicle (water, Fig 2B, 2D and 2E). We found that the antiviral activity of LPS-*Rs* was likely due to contaminating lipoproteins, as an ultrapure preparation of LPS-*Rs*, showed no antiviral activity (S2C Fig). Moreover, other known TLR2 agonists, including the diacylated lipopeptide Pam_2_CSK_4_, which binds TLR2/6 heterodimers, did not decrease RVFV infection in neurons (Fig 2E). We performed the screen in U2OS cells and found that the same two TLR2 ligands also decreased infection in these cells (Fig 2C). We investigated the antiviral activity of Pam_3_CSK_4_ as this ligand had defined concentrations of lipopeptide (unlike LPS-*Rs)*, and could be compared to Pam_2_CSK_4_, a similar molecule with no antiviral effect (S3 Fig).

### Pam_3_CSK_4_ is antiviral against *Phenuiviridae* and *Peribunyaviridae in vitro*

**Fig 3:**
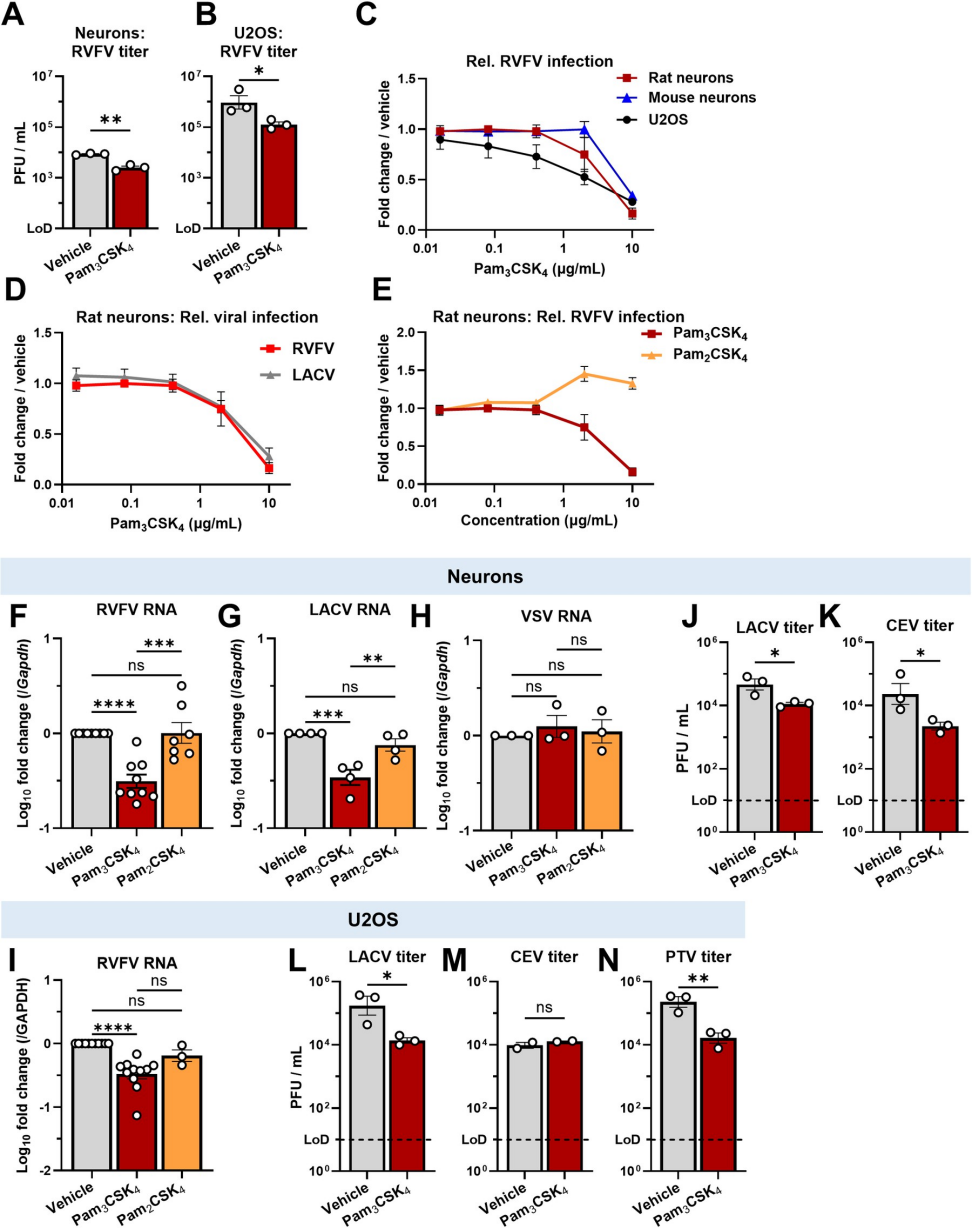
Pam_3_CSK_4_ is antiviral against bunyaviruses *in vitro*. **(A,B)** Neurons or U2OS were treated with vehicle (water) or 10μg/mL Pam_3_CSK_4_ prior to RVFV infection (neurons: MOI 0.1, U2OS: MOI 1). Supernatants were collected at 15hpi and titers were determined by plaque assay. LoD = limit of detection. *P = .0373, **P = .0015 **(C)** Rat (red squares) or mouse cortical neurons (blue triangles), or U2OS (black circles) were treated with the indicated dose of Pam_3_CSK_4_ for 4h before infection with RVFV (Neurons: MOI 0.3, U2OS: MOI 1). At 24hpi RVFV infection was determined by immunofluorescence automated microscopy and automated analysis. n = 3 (neurons) or 4 (U2OS). IC50 values were determined by nonlinear regression. **(D)** Rat neurons were treated as in (C), and infected with RVFV (MOI 0.3) or LACV (MOI 0.01) for 24h. Data acquired and presented as in (C). **(E)** Rat neurons were treated with serial dilutions of vehicle or TLR2 ligands 4h before RVFV infection. Data acquired and presented as in (C). **(F-H)** Rat neurons were treated with vehicle or TLR2 ligands at 10 μg/mL for 4h before infection with RVFV (F, MOI 0.1, 24hpi), LACV (G, MOI 0.01, 24hpi), or VSV-GFP (H, MOI 0.1, 14hpi). qPCR was used to measure viral Nucleocapsid RNA relative to *Gapdh*. F: ****P < .0001, *** *P =* .0001; G: ***P = .001, ** *P =* .0073 **(I)** U2OS were treated as in F, and infected with RVFV at an MOI of 1. Infection quantified as in F. ****P < .0001 **(J-N)** Neurons or U2OS were treated with vehicle or Pam_3_CSK_4_ at 10μg/mL before infection with LACV (J: MOI 0.015, L: MOI 0.1, 15hpi), CEV (K: MOI 0.05, M: MOI 5, 24hpi), or PTV (N: MOI 2, 15hpi). Titers quantified and data presented as in (A). J: *P = .0279, K: *P = .0447, L: *P = .0244, M: ns *P* = .3209, N: **P = .0082. Bars and symbols represent mean, error bars = SEM. Statistical analyses were performed using student’s t test on log_10_ transformed titers (A,B,J-N), or a one-way ANOVA with Tukey’s multiple comparisons test (F-I).

We validated that Pam_3_CSK_4_ had antiviral activity against RVFV using plaque assays, dose titrations, and qPCR. For plaque assays, we added Pam_3_CSK_4_ four hours before RVFV infection, and washed off the drug and viral inoculum two hours after infection. Pam_3_CSK_4_ reduced titers from 8.7 x 10^3^ to 2.6 x 10^3^ PFU / mL at 16 hpi (Fig 3A). In U2OS, titers were reduced from 5.2 x 10^5^ to 1.0 x 10^5^ PFU / mL (Fig 3B). Further, we found that there was a dose-dependent antiviral effect in rat neurons and U2OS cells, as measured by microscopy (neuron IC50 = 3.8 μg/mL; U2OS IC50 = 2.1 μg/mL) (Fig 3B). We also tested mouse cortical neurons and observed antiviral activity with no cytotoxicity, although the potency was lower (IC50 = 8.6μg/mL) (Figs 3B and S2D). These data demonstrate that Pam_3_CSK_4_ is antiviral against RVFV in primary rodent neurons and a human cell line.

We next tested the breadth of Pam_3_CSK_4_ antiviral activity. We treated rat neurons with several doses of Pam_3_CSK_4_ four hours before infection with the *Peribunyavirus* La Crosse virus (LACV) and found that Pam_3_CSK_4_ also inhibited LACV (Fig 3D). This led us to test Pam_3_CSK_4_ against two additional related viruses: the *Peribunyavirus* California Encephalitis virus (CEV, *Orthobunyavirus encephalitidis*) in the same group as LACV, and the *Phenuivirus* Punta Toro virus-Balliet (PTV, *Phlebovirus toroense*) in the same group as RVFV. CEV causes rare cases of encephalitis in humans, while PTV causes febrile illness but not encephalitis [49,50]. By plaque assay and qPCR, we found that Pam_3_CSK_4_ decreased RVFV, LACV and CEV infection in neurons (Figs 3A, 3F, 3G, 3J, 3K, S2F–S2G). PTV was not tested in neurons because infection was poor. In U2OS, Pam_3_CSK_4_ had antiviral activity against RVFV, LACV, and PTV, but not CEV (Figs 3B, 3C, 3I, 3L–3N, S2H–S2J). We also tested whether an unrelated negative sense RNA virus, the *Rhabdovirus* vesicular stomatitis virus (VSV, *Vesiculovirus Indiana*,) was sensitive to Pam_3_CSK_4_ treatment. VSV RNA replication was unchanged by Pam_3_CSK_4_ treatment, and the IC50 of Pam_3_CSK_4_ against VSV was >10μg/mL (Figs 3F and S2E). This suggests that Pam_3_CSK_4_ can inhibit bunyaviruses in the *Phenuiviridae* and *Peribunyaviridae*, though not all RNA viruses.

### Pam_2_CSK_4_, a diacylated lipopeptide, is not antiviral against RVFV or LACV

We screened several putative TLR2 ligands, including Pam_2_CSK_4_, which differs from Pam_3_CSK_4_ by one palmitoyl group, yet only Pam_3_CSK_4_ and LPS-*Rs* showed antiviral activity (Figs 2E and S3). TLR2 ligands can engage distinct receptor complexes, as Pam_2_CSK_4_ binds TLR2/6 heterodimers [42,51], while Pam_3_CSK_4_ binds TLR2/1 heterodimers [52]. TLR2/1 and TLR2/6 complexes signal through the same adaptor proteins [53,54]. Therefore, we explored whether Pam_2_CSK_4_ could have been a false negative in the screen. However, we found that treatment of neurons or U2OS with Pam_2_CSK_4_ had no antiviral effect on RVFV, LACV, or VSV, as observed by qPCR and microscopy (Fig 3D–3H). These data suggest that TLR2/1 and TLR2/6 signaling may differ in neurons, or that the structural differences between Pam_3_CSK_4_ and Pam_2_CSK_4_ may directly contribute to antiviral activity.

### Pam_3_CSK_4_-induced inflammatory signaling is not sufficient to control RVFV infection

**Fig 4:**
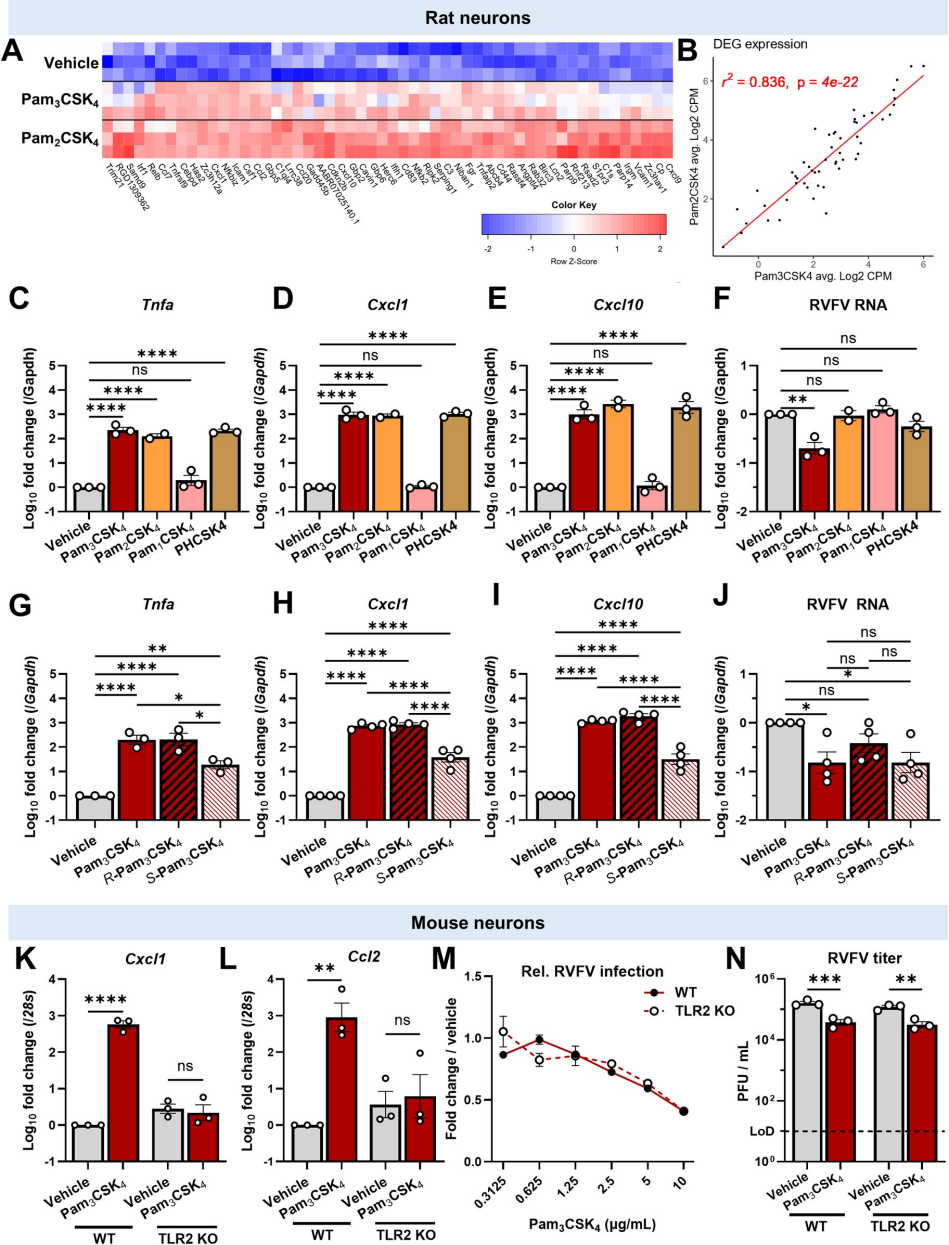
Pam_3_CSK_4_-induced inflammation is not antiviral in neurons. **(A)** Rat cortical neurons were stimulated with vehicle or 10μg/mL of Pam_3_CSK_4_ or Pam_2_CSK_4_ for 6h, and transcriptomic analysis was used to identify differentially expressed genes (≥2 Log_2_ fold change compared to vehicle, adjusted *P* ≤0.01). Heatmap shows hierarchical clustering of DEG. Rows are independent replicates. Color represents row Z score. **(B)** Linear regression of DEG expression after Pam_3_CSK_4_ or Pam_2_CSK_4_ stimulation. Genes from (A) are plotted by average Counts Per Million under each condition. **(C-E)** Relative expression of *Tnfa* (C), *Cxcl1* (D), and *Cxcl10* (E) in rat neurons stimulated with vehicle or 10μg/mL of lipopeptides for 4h. C: ****P < .0001; D: ****P < .0001; E: ****P < .0001. **(F)** Relative RVFV RNA in rat neurons pretreated with lipopeptides for 4h. Cells were treated as in C-E, and infected (MOI 0.1) for 24h. Data represented/analyzed as in C-E. **P = .0011. **(G-J)** Rat neurons were treated and analyzed as in (C-F), but with racemic or stereoisomeric Pam_3_CSK_4_. G: ****P< .0001, **P = .0043, Pam_3_CSK_4_ vs *S*-Pam_3_CSK_4_ *P = .0149, *R-*Pam_3_CSK_4_ vs *S*-Pam_3_CSK_4_ *P = .0144; H: ****P< .0001; I: ****P< .0001; J: vehicle vs. Pam_3_CSK_4_ *P = .0314, vehicle vs. *S*-Pam_3_CSK_4_ *P = .0317. **(K-L)** Relative expression of *Cxcl1* (K) or *Ccl2* (L) RNA in cortical neurons derived from wild type or TLR2 KO mice, stimulated with Pam_3_CSK_4_ for 4h. K: ****P< .0001; L: **P = .0016. **(M)** Wild type (black symbols, solid line) or TLR2 KO (empty symbols, dashed line) mouse neurons were treated with the indicated dose of Pam_3_CSK_4_ before RVFV infection (MOI 0.3). At 24hpi infection was measured by immunofluorescence automated microscopy and automated analysis. Infection normalized to vehicle treated neurons. n = 2. **(N)** WT or TLR2 KO mouse neurons were treated with 10μg/mL Pam_3_CSK_4_ for 4h, and infected with RVFV (MOI 0.1) for 15h. Titers were determined by plaque assay and log_10_ transformed for statistical analysis. **P = .0015, ***P = .0008. (C-N) Bars and symbol represent mean, error bars = SEM. Statistical analyses were performed using one-way ANOVA with Tukey’s multiple comparisons test (C-J), or two-way ANOVA with Šídák’s multiple comparisons test (K,L,N).

To compare the neuronal response to Pam_3_CSK_4_ and Pam_2_CSK_4_, we used RNA sequencing (RNAseq) and transcriptomic analysis. Time course qPCR studies of *Tnfa* and *Cxcl1* RNA levels after Pam_3_CSK_4_ stimulation revealed rapid induction of both genes, with *Tnfa* peaking at four hours and *Cxcl1* at eight hours (S4A and S4B Fig). Therefore, we stimulated neurons with vehicle, Pam_3_CSK_4_, or Pam_2_CSK_4_ for six hours and identified differentially expressed genes (adjusted *P* value of <0.01, and a Log_2_ fold change >2) compared to vehicle treated cells. In Pam_3_CSK_4_ stimulated cells, 24 genes were significantly upregulated (Fig 4A). In Pam_2_CSK_4_ stimulated cells, 53 genes were upregulated (Fig 4A). There were no significantly downregulated genes in either condition. Of the 24 Pam_3_CSK_4_ induced genes, 23 were also significantly induced by Pam_2_CSK_4_ (Fig 4A). Further, these genes were stimulated to similar levels: all differentially expressed genes were plotted by Counts per million (CPM) after treatment, and a linear regression was performed, demonstrating that the expression of Pam_3_CSK_4_ and Pam_2_CSK_4_-induced genes was highly correlated (Fig 4B, r^2^ = 0.836, p< 4 x 10^−22^). Upregulated genes were enriched for inflammatory cytokines and chemokines, including *Cxcl1* and *Cxcl10* (Fig 4A). Metascape pathway analysis [55] revealed that both treatments activated similar sets of pathways, and for both ligands, the most strongly enriched Gene Ontology (GO) term clusters were (inflammatory response) and (innate immune response) (S4C Fig). Thus, Pam_3_CSK_4_ and Pam_2_CSK_4_ induce similar transcriptional responses largely consisting of NF-κB-regulated genes in rat neurons. We confirmed that *Tnfa*, *Cxcl1*, and *Cxcl10* are induced by both treatments using qPCR (Fig 4C–4E). We also found that both treatments induced TNFα and CXCL10 in U2OS cells, though with a smaller fold change (S4F and S4G Fig). While these ligands do not canonically induce ISGs, we also tested if *Ifit2* was induced by Pam_3_CSK_4_ treatment at 4h or 24h. We saw no induction of *Ifit2* in neurons, or IFIT1 in U2OS cells (S4D and S4E Fig). These data show that Pam_3_CSK_4_ and Pam_2_CSK_4_ induce a similar set of NF-κB-dependent genes, and since only Pam_3_CSK_4_ is antiviral, our data suggests that the activity is likely independent of this pathway.

To directly test if Pam_3_CSK_4_ antiviral activity requires NF-κB signaling, we took advantage of two inhibitors of NF-κB activation [54]. IKK 16 is an inhibitor of multiple IkBa kinases (IKKs)[56] while TPCA-1 inhibits IKK2[57]. Both block the degradation of IκB, preventing NF-κB activation after an inflammatory stimulus. As expected, we observed blunted transcription of the NF-κB target genes *Tnfa* and *Cxcl1* in neurons that were treated with IKK 16 or TPCA-1 prior to Pam_3_CSK_4_ stimulation (S4H and S4I Fig). Despite this, neither inhibitor impacted the antiviral activity of Pam_3_CSK_4_, as RVFV RNA levels were significantly decreased by Pam_3_CSK_4_ in the presence of TPCA-1 or IKK 16 (S4J Fig). These data further suggest that Pam_3_CSK_4_ does not control RVFV through NF-κB-dependent transcriptional pathways.

### Structure-activity relationship

Since we observed little difference in gene expression but striking differences in antiviral activity between Pam_3_CSK_4_and Pam_2_CSK_4_, we set out to define a structure-activity relationship. Thus, we tested a more extensive panel of related lipopeptides. Pam_1_CSK_4_ has the same peptide motif as Pam_3_CSK_4_, but only one 15-carbon palmitoyl group [52] (S3 Fig). PHCSK_4_ has three hydrocarbon chains, but two ester-bonded hydrocarbon chains are bound directly to the oxygen in the ester group, rather than the carbon [52] (S3 Fig). Both Pam_1_CSK_4_ and PHCSK_4_ reportedly fail to activate TLR2[52,58]. However, when we stimulated rat neurons with Pam_3_CSK_4_, Pam_2_CSK_4_, Pam_1_CSK_4_, or PHCSK_4_ for 4h, we observed that Pam_3_CSK_4_, Pam_2_CSK_4_, and PHCSK_4_ induced inflammatory gene transcription (Fig 4C–4E). Despite this, only Pam_3_CSK_4_ decreased RVFV RNA (Fig 4F), suggesting that the number and composition of the hydrocarbon chains are important for antiviral activity. Next, we used enantiomers of Pam_3_CSK_4_ to test whether the orientations of the palmitoyl groups are determinants of antiviral activity. *R*-Pam_3_CSK_4_ has been reported to have a stronger interaction with TLR2/1 dimers than *S*-Pam_3_CSK_4_[43,59,60]. In concordance, qPCR showed that a four hour stimulation with *R-*Pam_3_CSK_4_ induced the transcription of *Cxcl1*, *Cxcl10*, and *Tnfa* like racemic Pam_3_CSK_4_, and better than the *S* isomer (Fig 4G–4I). Despite this, a four hour pretreatment with *R*-Pam_3_CSK_4_ failed to significantly decrease RVFV RNA replication at 24hpi, while *S*-Pam_3_CSK_4_ was as potent as racemic Pam_3_CSK_4_ (Fig 4J). Thus, the stimulation of inflammatory signaling was not correlated with Pam_3_CSK_4_ antiviral activity, which depended on the number and orientation of the hydrocarbon chains.

### Pam_3_CSK_4_ antiviral activity does not require TLR2

Although the preceding data suggested that Pam_3_CSK_4_ does not require inflammatory signaling for antiviral activity, this did not rule out a role for TLR2. Therefore, we cultured cortical neurons from TLR2 knock out (TLR2 KO) or wild type C57BL/6 mice [61]. We first tested whether the deletion of TLR2 prevented inflammatory signaling. We measured *Cxcl1* and C-C motif chemokine ligand 2 (*Ccl2*, also known as *Mcp-1)* levels by qPCR after a four hour Pam_3_CSK_4_ stimulation and found that Pam_3_CSK_4_ induced expression of these genes in wild type neurons, but this response was ablated in TLR2 KO neurons (Fig 4K and 4L). Next, we tested whether Pam_3_CSK_4_ had antiviral activity. In dose response microscopy studies, we found that Pam_3_CSK_4_ protected both WT and TLR2 KO neurons from RVFV infection (Wild type IC50 = 7.0μg/mL, TLR2 KO IC50 = 7.7μg / mL; Fig 4M). Pam_3_CSK_4_ pretreatment decreased RVFV titers from 1.6 x 10^5^ PFU / mL to 3.7 x 10^4^ PFU / mL in wild type neurons, and 1.2 x 10^5^ PFU / mL to 3.2 x 10^4^ PFU / mL in TLR2 KO neurons (Fig 4N). Similar patterns were observed for RVFV RNA, although the decrease was not significant in WT neurons due to variability (S5C Fig). We conclude that Pam_3_CSK_4_ has antiviral activity against bunyaviruses that is independent of TLR2 or downstream signaling.

### Pam_3_CSK_4_ inhibits RVFV entry by blocking viral fusion

**Fig 5:**
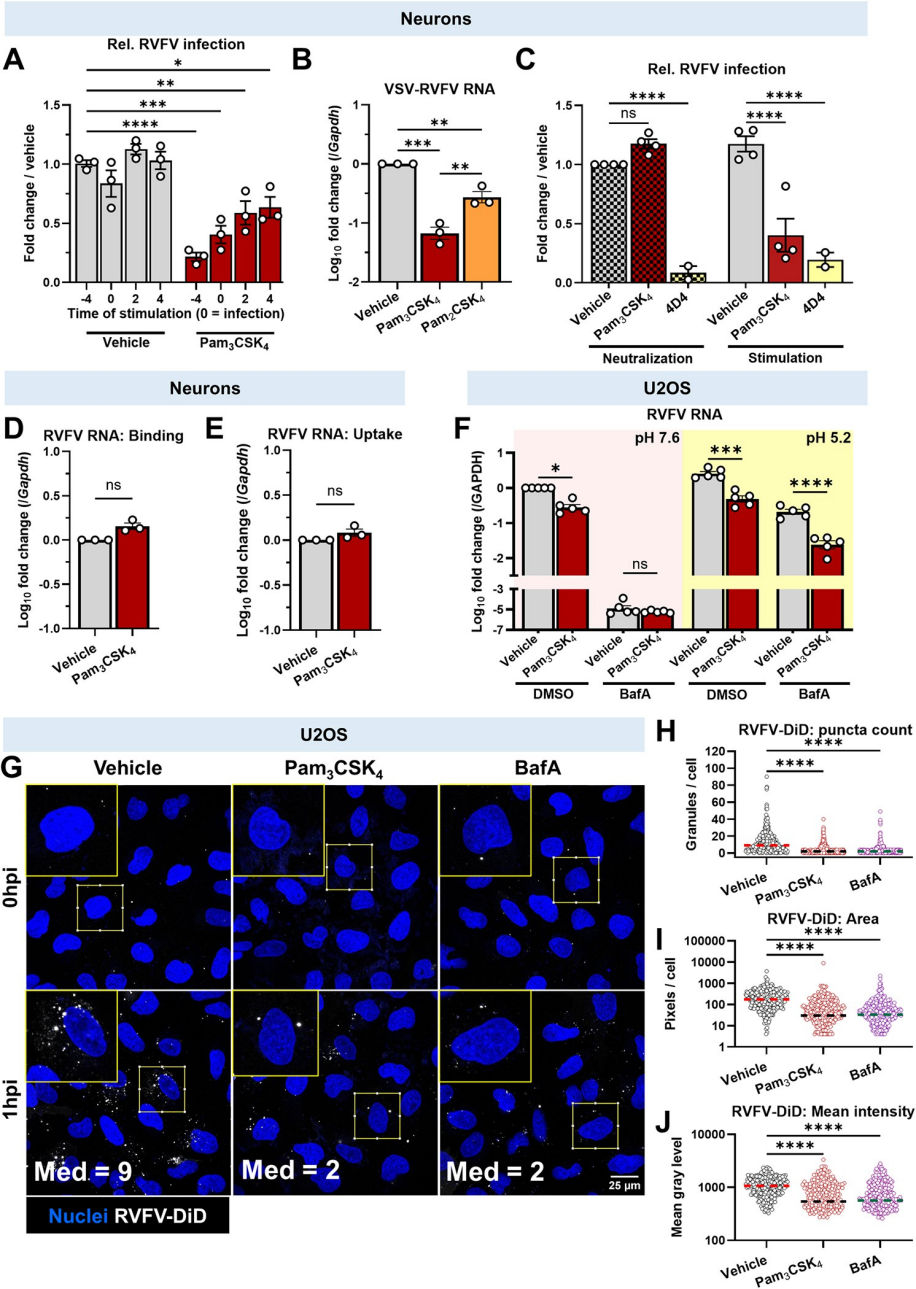
Pam_3_CSK_4_ blocks viral entry by reducing fusion. **(A)** Quantification of automated microscopy and analysis to detect RVFV infected rat neurons (MOI 0.3, 24hpi) when Pam_3_CSK_4_ (10μg/mL) or vehicle were added at the indicated timepoints (pre- or post-infection). Infection = time 0. Infection calculated relative to -4h, vehicle treated cells. *P = .0169, **P = .0069, ***P = .0002, ****P< .0001. **(B)** Quantification of VSV N RNA in rat neurons treated with 10μg/mL of TLR2 ligands 4h before VSV-RVFV infection (MOI 5, 14hpi). RNA levels measured by qPCR, relative to *Gapdh*. ***P = .0001, vehicle vs. Pam_2_CSK_4_ **P = .0061, Pam_3_CSK_4_ vs. Pam_2_CSK_4_ **P = .0042. **(C)** Quantification of automated microscopy and analysis of RVFV-infected rat neurons after Pam_3_CSK_4_ neutralization. Vehicle, Pam_3_CSK_4_, or 4D4 anti-RVFV Gn were either added to cells (stimulation, solid bars) or mixed with stock RVFV (neutralization, checked bars) for 4h. RVFV or neutralized RVFV was used to infect the cells at a MOI of 0.3 for 24h. ****P< .0001 **(D)** RVFV (MOI = 5) was bound to rat neurons at 15°C in the presence or absence of 10μg/mL Pam_3_CSK_4_. RVFV N RNA was measured by RT-qPCR, normalized to *Gapdh*, and set relative to vehicle. **(E)** RVFV was bound to neurons in the presence or absence of Pam_3_CSK_4_ as in (D), and cells were shifted to 37°C for 2h to allow viral uptake. Cells were trypsinized to remove remaining surface bound particles and RVFV RNA was measured, analyzed and shown as in (D). **(F)** Quantification of RVFV N RNA in U2OS following acid bypass assay. Cells were pretreated with Pam_3_CSK_4_ at 10μg/mL and bafilomycin A1 (0.05μM) or DMSO prior to RVFV binding at 15°C (MOI 0.2). Cells were pulsed with neutral (pink) or acidic (yellow) OptiMEM containing water or Pam_3_CSK_4_, and then cells were incubated for 24h in the presence or absence of BafA. *P = .0100, ***P = .0006, ****P< .0001**. (G)** Confocal microscopy showing RVFV-DiD puncta (white) in U2OS cells (nuclei stained blue). 60x magnification, scale bar = 25μm. Z-stacks were acquired and are shown as maximum projections. Images representative of five sites per condition, n = 4. **(H-J)** MetaXpress software was used for automatic quantification of 1h timepoint from (G), showing **(H)** the number of DiD puncta per cell, **(I)** the total area of DiD per cell, (**J**) the average intensity of DiD puncta within each cell. Each dot represents one cell. Vehicle: 322 cells analyzed; Pam_3_CSK_4_: 385 cells analyzed; BafA: 385 cells analyzed. For (I and J), cells with 0 puncta are not plotted due to the log scale of the Y axis. Dotted lines represent median of compiled experiments. ****P< .0001. For A-F, bars represent mean, error bars = SEM. Statistical analyses were performed using two-way ANOVA with Dunnett’s multiple comparisons test (A), one-way ANOVA with Tukey’s multiple comparisons test (B,H-J), two-way ANOVA with Šídák’s multiple comparisons test (C, F), or Welch’s t test (D,E).

As our data suggested that Pam_3_CSK_4_-induced inflammatory signaling was not antiviral, we sought to identify the step in the viral lifecycle inhibited by Pam_3_CSK_4_. We began by performing a time of addition assay, where Pam_3_CSK_4_ was added to neurons four hours before infection (t = -4), at the time of infection (t = 0), or two-four hours afterwards, and quantified infection by microscopy at 24 hpi. We found that the potency of Pam_3_CSK_4_ treatment decreased over time, with infection reduced to 22% of control at t = -4, 40% at t = 0, 59% at t = +2, and 64% at t = +4, suggesting that Pam_3_CSK_4_ may act on an early stage of viral infection (Fig 5A). Our plaque assays also supported this, as Pam_3_CSK_4_ decreased RVFV titers even though it was washed off two hours after infection (Fig 3A and 3B).

To test if Pam_3_CSK_4_ inhibits RVFV entry, we measured the antiviral activity of Pam_3_CSK_4_ against VSV-RVFV, a recombinant VSV expressing the RVFV glycoproteins Gn and Gc from the clinical isolate RVFV ZH501[1,15,38]. Using VSV-RVFV, we tested whether Pam_3_CSK_4_ targets RVFV glycoprotein-dependent entry. Pam_3_CSK_4_ pretreatment decreased VSV-RVFV replication in neurons and U2OS (Figs 5B and S5B). This was independent of TLR2, as similar inhibition was observed in wild type or TLR2 KO mouse neurons (S5D–S5F Fig). This demonstrates that Pam_3_CSK_4_ blocks RVFV glycoprotein-dependent entry. We noted that Pam_2_CSK_4_ also reduced VSV-RVFV infection, although it was significantly weaker than Pam_3_CSK_4_ (Fig 5B).

We then performed a series of experiments to define the step in the entry process that is blocked by Pam_3_CSK_4_, using RVFV MP-12. First, we tested whether Pam_3_CSK_4_ directly neutralizes RVFV virions. We mixed 10μg/mL of Pam_3_CSK_4_, vehicle, or the RVFV-neutralizing monoclonal antibody 4D4[62] with 6.7 x 10^6^ PFU of RVFV (neutralization condition) and incubated at 37°C for four hours. We then used this mixture to infect the cells at a final MOI of 0.4, which diluted the Pam_3_CSK_4_ to 0.1μg/mL, below the active concentration. We compared this neutralization condition to our established Pam_3_CSK_4_ pretreatment (stimulation condition), where we treated the cells with 10μg/mL Pam_3_CSK_4_ for four hours, and then infected with 0.4 MOI of virus. At 24 hours, we quantified infection by microscopy, and observed that Pam_3_CSK_4_ failed to neutralize RVFV but was antiviral when preincubated with the cells (Fig 5C). 4D4 prevented infection under both conditions [62] (Fig 5C). These findings suggest that Pam_3_CSK_4_ does not directly inactivate or neutralize virions but works at a step downstream.

To test whether Pam_3_CSK_4_ impacts RVFV attachment to cells, we used qPCR to measure cell-associated virions. We treated either neurons or U2OS cells with Pam_3_CSK_4_ or vehicle and infected with RVFV at 15°C, which allowed for binding but prevented internalization. After one hour we washed the cells to remove unbound virus and measured bound viral RNA by qPCR. As a positive control we trypsinized the cells to remove bound virions, and indeed, we were able to strip bound virus (S6A and S6C Fig). Pam_3_CSK_4_ did not change the amount of viral RNA bound to cells (Figs 5D, S6A and S6C).

Next, we investigated whether Pam_3_CSK_4_ treatment decreased viral internalization. After binding RVFV at 15°C, we shifted neurons or U2OS to 37°C for two hours to allow endocytic uptake to resume and virions to become internalized. We removed the virions that remained on the surface with trypsinization, and quantified internalized viral RNA by qPCR. We found that trypsinization modestly reduced the levels of RVFV RNA, suggesting that virions were efficiently internalized by neurons at this time point (S6B Fig). We observed no difference in internalized RVFV RNA in the presence of Pam_3_CSK_4_ (Figs 5E, S6B and S6D). Together, these assays suggest that Pam_3_CSK_4_ inhibits RVFV entry at a step following viral attachment and uptake.

The final step in the entry process involves the fusion of the virion and endosomal membranes [13]. Thus, we tested whether RVFV fusion is inhibited. First, we performed an acid bypass assay, which bypasses endocytosis and forces fusion at the plasma membrane [13]. This allowed us to determine whether Pam_3_CSK_4_ blocked a step in endocytic entry or blocked viral fusion directly. To validate the assay, U2OS cells were pretreated with vehicle or the endosomal acidification inhibitor bafilomycin A1 (BafA) and the cells were then cooled to 15°C, to allow viral binding but not uptake. RVFV was bound for one hour and then the cells were pulsed for 10 minutes with media at neutral pH (pH 7.6) or acidic pH (pH5.2), as RVFV fusion is induced at pH 5.2[13,63]. Cells were washed and incubated in media at neutral pH for 24h and viral RNA was measured by qPCR. As expected, we found that BafA treatment blocked infection at pH 7.6 since endosomal entry was inhibited (Fig 5F). In contrast, BafA did not block infection in acid-washed cells, as fusion occurred at the cell surface (Fig 5F). In parallel, we treated the cells with Pam_3_CSK_4_, which inhibited RVFV infection in DMSO treated cells (Fig 5F). We found that Pam_3_CSK_4_ also decreased RVFV infection in acid-washed, BafA-treated cells, suggesting that Pam_3_CSK_4_ inhibited RVFV fusion at the cell surface (Fig 5F).

We performed a lipid dye dequenching assay [64,65] to verify that Pam_3_CSK_4_ blocks viral fusion. RVFV virions were labelled with the lipophilic fluorescent dye, 1,1’-Dioctadecyl-3,3,3’,3’-Tetramethylindodicarbocyanine Perchlorate (DiD, 30μM). At this concentration, the virions self-quench and do not fluoresce. However, once virions fuse with the larger endocytic membrane, the dye diffuses and can be measured by fluorescence microscopy. Thus, quantification of DiD puncta is a measurement of viral fusion. We again validated the assay using BafA, which blocks viral fusion. DiD labeled RVFV (RVFV-DiD, MOI 15) was bound to U2OS cells in the presence of vehicle or BafA at 15°C to allow binding but not uptake. Cells were fixed after binding (t = 0) or shifted to 37°C for one hour to allow viral uptake and fusion. Few puncta were detected at t = 0 (Fig 5G), demonstrating that prior to fusion, RVFV-DiD particles do not fluoresce (Fig 5G). In contrast, after incubation at 37°C, most cells showed multiple puncta in vehicle treated conditions (Fig 5G). As expected, BafA significantly decreased the number of puncta per cell, as well as the median area of DiD positive pixels per cell and the median intensity of puncta (Fig 5H–5J). Upon treatment with Pam_3_CSK_4_, we also observed decreases in number of puncta per cell, the median area of DiD positive pixels per cell, and the median intensity of puncta (Fig 5H–5J), demonstrating again that Pam_3_CSK_4_ blocks viral fusion. We performed a parallel experiment in primary neurons (S7 Fig). Although fewer puncta were observed per cell in control treated neurons (median = 2 in neurons vs. median = 9 in U2OS cells), the number, intensity, and area of DiD puncta were decreased in neurons after BafA or Pam_3_CSK_4_ treatment (S7A–S7D Fig). These data further demonstrate that Pam_3_CSK_4_ inhibits RVFV fusion.

### Pam_3_CSK_4_ protects mice against encephalitic RVFV infection

**Fig 6:**
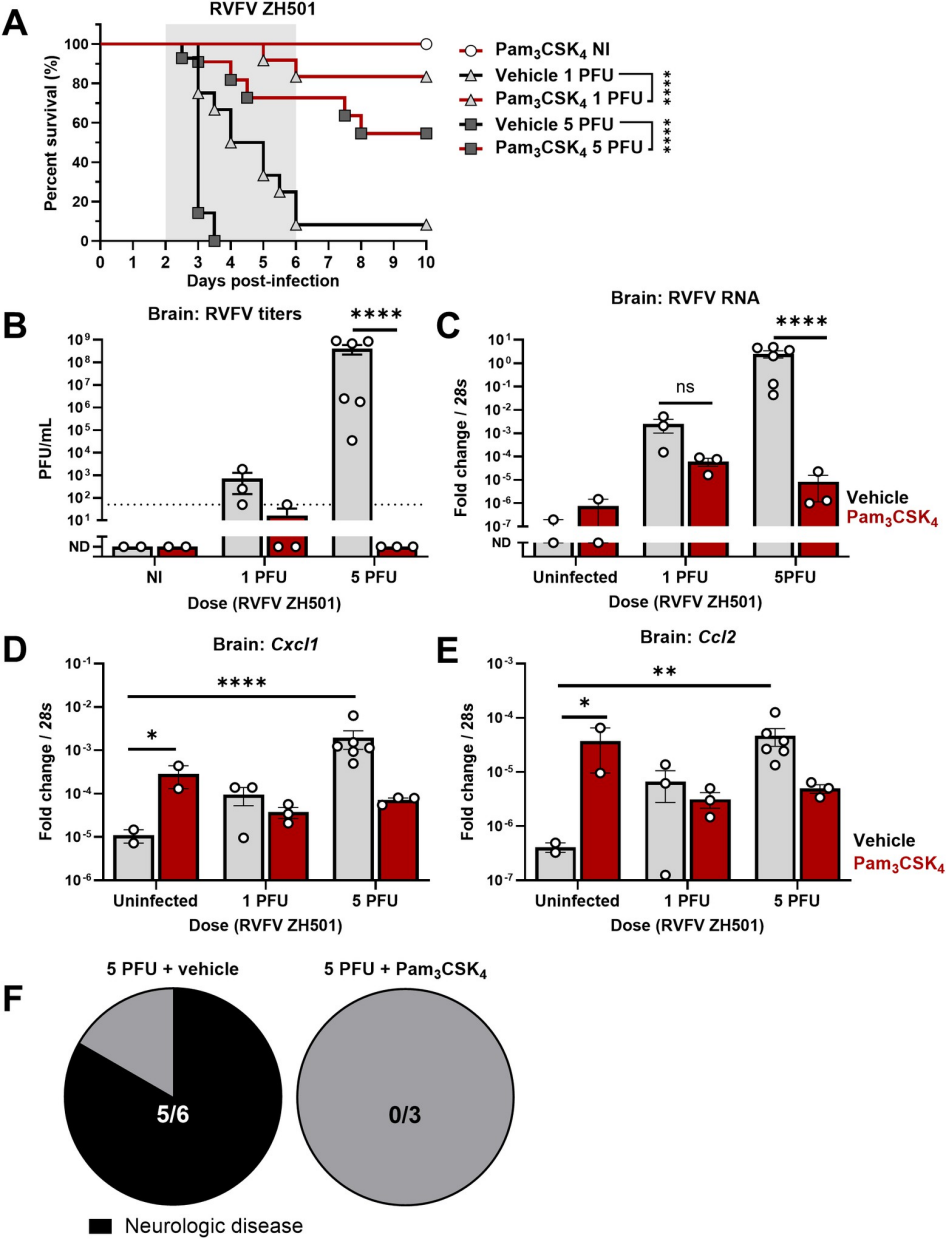
Pam_3_CSK_4_ protects mice from intracerebral RVFV infection. **(A)** Survival curve of 3-6-week-old C57BL/6 mice infected intracranially with 1 or 5 PFU of RVFV ZH501 (triangles or squares, respectively) with or without 100 μg of Pam_3_CSK_4_ administered simultaneously (Black lines = vehicle, red lines = Pam_3_CSK_4_). Gray shading indicates period where infected mice are expected to succumb. n = 11-14/group infected; n = 2/group not infected (NI). ****P< .0001 **(B)** Mice were infected with RVFV with vehicle or Pam_3_CSK_4_ as in (A). At 3 dpi, RVFV titers in the mouse brain were determined by plaque assay. ****P< .0001 **(C)** RNA levels in the mouse brain were determined by qPCR, relative to mouse 28s RNA, from the same mice as in (B). ****P< .0001 **(D-E)** *Cxcl1* (D) and *Ccl2* (E) RNA levels in the mouse brain at 3dpi, relative to mouse 28s RNA. D: *P = .0162, ****P< .0001; E: *P = .0151, **P = .0019. **(F)** Pie chart representing the proportion of mice with neurological disease at 3 dpi, from the 5 PFU group. Black = neurologic disease. All bars represent mean, error bars = SEM. Statistical analyses performed using Mann-Whitney U test (A), 2-way ANOVA with Šídák’s multiple comparisons test (D,E), or two-way ANOVA with Tukey’s multiple comparisons test (B,C).

As Pam_3_CSK_4_ decreased RVFV infection in primary neurons, we tested whether Pam_3_CSK_4_ was protective in a mouse model of RVFV encephalitis. Three to six week-old C57BL/6 mice were inoculated intracranially with RVFV ZH501, mixed with vehicle or Pam_3_CSK_4_. This route of inoculation allowed us to control the timing and input of virus into the central nervous system. 10% of mice infected with 5 PFU survived to day 3, and 5% of mice infected with 1 PFU survived to 6 dpi, demonstrating that mice are reliably infected at these doses (Fig 6A). However, Pam_3_CSK_4_ was protective, with greater than 50% of treated mice surviving 5 PFU and 80% surviving 1 PFU, while all mice treated with Pam_3_CSK_4_ alone survived (Fig 6A). Consistent with this drastic increase in survival, we found that Pam_3_CSK_4_ treatment decreased viral replication in the brain by more than 5 logs when mice were infected with 5 PFU (Fig 6B and 6C). At this timepoint (3 dpi), five out of six mice that received 5 PFU of RVFV plus vehicle showed signs of severe disease, compared to zero out of three Pam_3_CSK_4_ treated, infected mice (Fig 6F). Titers were also significantly decreased in the liver at day 3, suggesting that Pam_3_CSK_4_ limited spread of the virus (S8A Fig).

We also monitored NF-κB-dependent inflammatory responses in the brain at 3 days. RVFV infection with 1 PFU induced *Cxcl1* approximately 10-fold while 5 PFU induced these transcripts by ~100-fold, consistent with previous studies of RVFV encephalitis [66,67] (Fig 6D and 6E). In uninfected animals, Pam_3_CSK_4_ induced *Cxcl1* approximately 10-fold, similar to the 1 PFU infection and reflecting our RNAseq results. However, Pam_3_CSK_4_ treatment alleviated inflammation caused by severe RVFV infection (5 PFU), as we observed ~10-fold decreased *Cxcl1* levels relative to infected, vehicle-treated animals (Fig 6D and 6E). We also monitored *Ccl2* mRNA, as this cytokine has also been associated with RVFV encephalitis and was differentially expressed in our RNAseq experiments [66,67]. We observed a similar pattern, as Pam_3_CSK_4_ induced *Ccl2* expression in uninfected animals, but decreased *Ccl2* levels in the context of 5 PFU infection. Hematoxylin and eosin staining of mouse brains and livers showed that Pam_3_CSK_4_ treatment reduced hemorrhages caused by RVFV in the liver (S8B Fig). Immunofluorescence for RVFV nucleoprotein also showed that Pam_3_CSK_4_ decreased viral replication in the brain and liver at 3 dpi (S8C Fig). Altogether, Pam_3_CSK_4_ protected mice from neuronal RVFV infection, and reduced spread from the central nervous system to peripheral tissues.

## Discussion

RVFV infection leads to acute disease, but in a subset of individuals, severe disease including delayed-onset meningoencephalitis can result in long term neurologic sequelae or death [6,21]. As these symptoms present in the later stages of infection, there is potentially a window of time to deliver therapeutics. However, there are no approved treatments for humans and little is known about preventing RVFV infection in neurons, a vulnerable and essential cell type. We thus explored RVFV infection of primary neurons as these are the natural target cells infected in the human and rodent central nervous system [21]. Previous studies found that neurons have an altered response to type I IFN stimulation, which can leave them vulnerable to RNA virus infection [25–28]. Indeed, IFNα, IFNβ, or IFNγ-stimulated neurons were not strongly protected from RVFV infection.

We sought to identify alternative innate immune agonists with antiviral activity. Of the 75 ligands we tested, five were antiviral without cytotoxicity in primary neurons, including two TLR2 ligands. However, several other TLR2 ligands had no antiviral activity, suggesting that TLR2 stimulation is not sufficient to inhibit infection. We focused on the well-defined synthetic lipopeptides Pam_3_CSK_4_ and Pam_2_CSK_4_ which are structurally similar but had divergent effects on infection: Pam_3_CSK_4_ was antiviral while Pam_2_CSK_4_ was not. Validation experiments recapitulated this pattern across multiple cell types and species. Further, Pam_3_CSK_4_ limited infection with other members of the *Peribunyaviridae* and *Phenuiviridae*, but not the unrelated negative sense *Rhabdovirus* VSV. In U2OS, Pam_3_CSK_4_ had no antiviral activity against CEV, which may be related to the high MOI of 5 needed for robust infection in these cells. Overall, Pam_3_CSK_4_ may have a specific antiviral effect against viruses within the *Phenuiviridae* and *Peribunyaviridae* families. Other families of the *Bunyavirales*, and other encephalitic viruses remain untested.

As TLR2 canonically activates NF-κB, and Pam_3_CSK_4_ reportedly antagonizes Hepatitis B virus through NF-κB signaling [68], we investigated whether RVFV was similarly controlled. Previous work has shown that TLR2 activating lipopeptides induce inflammatory pathways in the brain, but these responses have largely been attributed to microglia or astrocytes [69–71]. Thus, we used transcriptomics to define the genes induced by Pam_3_CSK_4_ and Pam_2_CSK_4_ in primary neurons. We found that both ligands induced canonical NF-κB-dependent inflammatory pathways, demonstrating that neurons respond to TLR2 ligands. It was not surprising that these responses were alike, as TLR2/1 and TLR2/6 heterodimers signal through the same adaptor proteins [53]. Moreover, these findings suggested that the antiviral effect of Pam_3_CSK_4_ is not mediated by NF-κB-induced gene expression, which was also supported by NF-κB inhibitor experiments. In fact, we showed that the antiviral activity of Pam_3_CSK_4_ is independent of TLR2, as TLR2 knock out neurons were protected by Pam_3_CSK_4_.

To further define the structural requirements for antiviral activity, we took advantage of a panel of molecules related to Pam_3_CSK_4_. PHCSK_4_, a similar molecule with a modified backbone, activated inflammatory gene transcription but had no antiviral activity against RVFV, which indicates that the backbone of the hydrocarbon chains is a determinant of antiviral activity. Next, we tested stereoisomers of Pam_3_CSK_4_, as the *R* isomer binds more strongly to TLR2/1 than the *S* isomer [43,72]. Although *S*-Pam_3_CSK_4_ stimulated chemokine transcription less potently than *R*-Pam_3_CSK_4_, it had a stronger antiviral effect, demonstrating that the orientation of the molecule matters for the control of infection.

Pam_3_CSK_4_ is cationic, and has been shown to impact viral binding to target cells in some circumstances [73]. Pam_3_CSK_4_ increases respiratory syncytial virus (RSV), measles virus, human metapneumovirus, and HIV binding and infection in primary airway epithelial cells and lymphoid cells, potentially through enhancing binding to glycosaminoglycan attachment factors [73]. Furthermore, this activity is specific and independent of TLR2, as Pam_2_CSK_4_ does not impact RSV infection [73]. As these effects were attributed to a change in surface binding in the presence of Pam_3_CSK_4_, we tested whether Pam_3_CSK_4_ impacts bunyavirus binding to target cells.

Unlike prior studies we found that Pam_3_CSK_4_ did not alter RVFV binding or uptake in target cells. However, by comparing VSV and VSV-RVFV infection, we showed that Pam_3_CSK_4_ blocked viral entry. Indeed, we found that Pam_3_CSK_4_ blocked viral fusion both in endosomes and at the plasma membrane, suggesting specific interactions independent of the compartment. We infer that Pam_3_CSK_4_ does not prevent endosomal acidification, as RVFV and VSV rely on a similarly low pH but Pam_3_CSK_4_ had no activity against VSV [18,63,74]. As our data show that the palmitoyl chains of Pam_3_CSK_4_ are important for antiviral activity, these hydrophobic groups may interact with the host and/or viral membrane to disrupt the function of the Gc fusion peptide. It has been found that the addition of lipid groups to some fusion-inhibiting peptides can enhance antiviral activity by increasing peptide concentrations on the membranes where fusion occurs [11,75]. We suggest that the palmitoyl groups of Pam_3_CSK_4_ have a similar function.

Amphipathic fusion inhibitors, which intercalate into virion membranes and irreversibly inactivate them, inhibit several enveloped viruses including RVFV [19,76,77]. Structurally, these molecules resemble a wedge, with narrow hydrophobic domains linked to bulky polar regions. The hydrophobic domains insert into viral membranes, stabilizing the pre-fusion curvature of the viral membrane [19,76]. While the Pam_3_CSK_4_ shares some structural similarities with this class of inhibitors, Pam_3_CSK_4_ does not irreversibly inactivate RVFV particles, demonstrating a distinct mechanism (Fig 5C).

Lastly, we tested whether Pam_3_CSK_4_ had antiviral activity *in vivo* in the central nervous system. We found a robust antiviral effect in the mouse brain, as the delivery of Pam_3_CSK_4_ reduced viral titers by several logs, and pathogenesis was dramatically reduced. RVFV encephalitis was highly inflammatory in the mouse brain at three dpi. Pam_3_CSK_4_ treated, infected mice had less inflammatory gene expression than infected, untreated mice, likely due to the reduction in viral replication. In addition to the direct effect of Pam_3_CSK_4_ on viral fusion in neurons, it is possible that TLR2-expressing cells responded to Pam_3_CSK_4_. Whether this is protective or pathogenic is unclear. It is known that TLR2 signaling in the brain can activate microglia and astrocytes, and recruit lymphocytes [70,78,79], and thus it is possible that these responses are indirectly impacting infection or pathogenesis. Further work is required to explore the impact of Pam_3_CSK_4_ on diverse cell types during severe neurologic bunyaviral infection. As we showed that *S*-Pam_3_CSK_4_ had limited inflammatory properties but retained antiviral activity, this stereoisomer could decouple these effects. We suggest that *S-*Pam_3_CSK_4_ would block viral fusion without inflammatory responses which may be beneficial. One challenge in using fusion inhibitors to treat acute infections is time, as they are most effective when present early in infection. However, RVFV meningoencephalitis is a late-presenting stage of disease, and fusion inhibitors may be useful in preventing infection in the brain. While it is unknown whether Pam_3_CSK_4_ can be used therapeutically in the brain, or whether it has anti-encephalitic activity when delivered systemically, these questions should be addressed in future studies. It will be important to test whether peripherally-delivered Pam_3_CSK_4_ can enter the brain, as delivery into the central nervous system is a challenge. However, encephalitis can result in blood-brain barrier permeability, which may enable Pam_3_CSK_4_ to enter.

In sum, this work describes a role for Pam_3_CSK_4_ as an inhibitor of viral fusion, which may have value in treating neurologic bunyavirus infections.

## Materials and methods

### Ethics statement

All mouse experiments were approved by the University of Pittsburgh Institutional Animal Care and Use Committee, protocol #23083513. No human participants or samples were used in this work.

### Cell cultures

Primary rat cortical neurons were isolated from E18 Sprague Dawley IGS pups (Charles River) by the Penn Medicine Translational Neuroscience Core as previously described [80]. Mouse cortical neurons were isolated from C57BL/6 (Charles River) or TLR2 KO (Jackson Laboratory RRID:IMSR_JAX:004650) embryos at E15.5-E16.5. After dissection in cold 1x PBS, cortices were resuspended and mechanically dissociated in room temperature OptiMEM (ThermoFisher) supplemented with 1% GlutaMAX (Invitrogen 3505006). Neurons were plated at 9.6 x 10^4^ cells/cm^2^ on plates or glass coverslips coated with 0.25mg/mL Poly-L-Lysine hydrobromide (Biosynth OKK-3056 or Electron Microscopy Sciences 72292). Rat neurons were plated and maintained in neurobasal medium (Invitrogen 21103049) with 2% B-27 (Invitrogen 12587010), 33mM glucose (Millipore-Sigma G8769), 40mM HEPES (Invitrogen 15630080), and 1% penicillin/ streptomycin (P/S, Invitrogen 15140122). Mouse neurons were plated in neurobasal medium with 10% heat-inactivated horse serum (Invitrogen 16050122), 1% pyruvate (Invitrogen 11360070), 33mM glucose, and 37.5mM sodium chloride. After four hours, the media was changed to neurobasal plus 2% B-27, 33mM glucose, 1% GlutaMAX, 37.5mM sodium chloride, 1% P/S, and 40mM HEPES. All neurons were cultured in humidified chambers at 37°C for 7 days before use, with 40% of the media being replaced on day 4. All neuron media was conditioned at 37°C and 5% CO_2_ before use. All neuron replicate experiments were performed on independent preparations of neurons. U2OS osteosarcoma cells (ATCC HTB-96) and Baby Hamster Kidney cells (BHK-21, ATCC CCL-10) were maintained in DMEM with 10% heat inactivated fetal bovine serum (Millipore-Sigma TMS-013-B), 1% P/S and 1% GlutaMAX.

### Drugs and treatments

Universal IFNα was purchased from Fisher scientific (112002), while rat IFNα and rat IFNβ were purchased from PBL Assay Science (13100–1, 13400–1) and rat IFNγ was from Peprotech (400–20). For screening, 75 innate immune ligands were sourced from Invivogen. For follow-up studies, Pam_3_CSK_4_ (tlrl-pms), Pam_2_CSK_4_ (tlrl-pm2s-1), LPS-*Rs* (tlrl-rslps), and LPS-*Rs* ultrapure (tlrl-prslps) were repurchased from Invivogen. Pam_3_CSK_4_ (L2000), Pam_1_CSK_4_ (L2011), PHCSK_4_ (L2032), *R-*Pam_3_CSK_4_ (L2048), and *S-*Pam_3_CSK_4_ (L2049) were purchased from EMC Microcollections. All IFNs and immune ligands were resuspended in water, and TLR2 ligands were used at 10μg/mL unless otherwise noted. TPCA-1 was purchased from Sigma-Aldrich (T1452), diluted in DMSO, and used at 6 μM. IKK 16 was purchased from Santa Cruz Biotechnology (sc-204009), diluted in DMSO, and used at 3 μM. Bafilomycin A1 was purchased from Sigma Aldrich (B1793), diluted in DMSO, and used at 0.05μM.

### Viral stocks

Rift Valley fever virus MP-12 (RVFV), La Crosse virus-Original (LACV), Vesicular Stomatitis Virus-green fluorescent protein (VSV-GFP), VSV expressing RVFV glycoprotein (VSV-RVFV-GFP, gift of Sean Whelan), were propagated in BHK-21 cells. Five amino acid substitutions are present in VSV-RVFV Gn/Gc relative to RVFV ZH501: Q232L, G566D, V602I, A640D, and Q983R. California encephalitis virus BFS-283 (CEV) was propagated on C6/36 cells, and Punta Toro virus Balliet (PTV) was propagated on LLC-MK2 cells. For all viruses, supernatant was clarified by spinning at 1000 x g for 5 minutes, and was then aliquoted. For DiD fusion experiments, clarified RVFV was passed through a 0.22μm filter, and pelleted through 20% sucrose by ultracentrifugation (29,000rpm for 2h on a Beckman Coulter SW-32 Ti rotor). Virus was resuspended in 25mM HEPES-buffered saline by shaking overnight at 4°C. Virus was diluted to 2mg/mL, and stained with 1,1’-Dioctadecyl-3,3,3’,3’-Tetramethylindodicarbocyanine Perchlorate (DiD, ThermoFisher Scientific D307) at 30μM for 1hr. Free dye was separated by Sephadex G-25 chromatography (Millipore-Sigma G25150). Virulent RVFV strain ZH501 was generated from reverse genetics plasmids [81] and provided to the Hartman laboratory by B. Miller (CDC, Ft. Collins, CO) and S. Nichol (CDC, Atlanta, GA). RVFV ZH501 was propagated on Vero E6 cells according to the protocol in McMillen, *et al* [82]. All work with virulent RVFV (strain ZH501) was performed in biosafety level 3 in the University of Pittsburgh Regional Biocontainment Laboratory (RBL) following safety precautions explained in McMillen, *et al* [82]. The University of Pittsburgh RBL is registered with the U.S. Department of Agriculture and the Centers for Disease Control and Prevention for work with virulent RVFV.

### Immunofluorescence

Cells were fixed with 4% formaldehyde for 10 minutes at room temperature, then washed three times with PBS to remove formaldehyde. Cells were then blocked and permeabilized in blocking buffer (PBS plus 2% Bovine Serum Albumin (BSA) and 0.1% Triton-X100) at room temperature for 1 hour. Primary antibodies were diluted in blocking buffer and used to stain cells at 4°C overnight. To stain MAP2, we used chicken (Abcam ab5392) or rabbit (BioLegend 840601) polyclonal antibodies diluted 1:3000. Viruses were detected using anti-RVFV Gn (clone 4D4), or anti-LACV G1 (Clone 807.31+807.33). After primary antibody binding, cells were washed 3 times with PBS. Fluorophore-conjugated, species-specific secondary antibodies and Hoechst 33342 were then diluted in blocking buffer and added to cells for a 1-hour incubation at room temperature. Cells were washed again 3 times with PBS. For automated microscopy, cells were imaged on a Molecular Devices ImageXpress Micro 4 imaging system. Images were quantified using Molecular Devices MetaXpress (version 6) modules including Cell Scoring, Multiwavelength Cell Scoring, and Granularity. For confocal microscopy experiments, the final PBS wash was replaced with H_2_O and coverslips were mounted in Vectashield (Fisher Scientific NC9265087) and sealed with clear nail polish. Coverslips were imaged on a laser scanning Leica TCS SPE-II with 40x or 63x objective lenses with 1.5x zoom and a pinhole of 1 at the highest wavelength. Sites were selected based on the nuclear channel alone, and the same settings were used within each experiment. The following fluorophores were used: Hoechst 33342, AlexaFluor 488, AF594, Cy5, and DiD (excitation at 633 nm). Images were captured at a resolution of 1024x1024 pixels. Images were equally levelled for display using ImageJ, and granularity was analyzed using MetaXpress, with a minimum granule size of 3μm and a maximum size of 15μm.

For hematoxylin and eosin (H & E) staining and immunofluorescent imaging of tissues, samples underwent cryopreservation through 24 hour incubations in 20% sucrose in PBS, then 40% sucrose in PBS prior to freezing in mounting optimal cutting temperature (OCT) compound (Fisher) and long-term storage at -80°C. Tissues were sliced to 5 μm sections using the Histocore Autocut CryoStar X7-CryoStat (ThermoFisher Scientific) and stored at -80°C until staining was performed. Tissues were stained by standard H & E methods and images were taken at 20x magnification on an Olympus CX41 microscope with a Levenhuk microscope digital camera (M base series). For immunofluorescent imaging, cryo-sections were rehydrated with PBS containing 0.5% bovine serum albumin (PBB), and then blocked in 5% normal goat serum in PBB. The tissues were washed, then permeabilized in 0.1% Triton X-100 detergent for 15 minutes. The tissues were washed, then probed with custom rabbit anti-RVFV nucleoprotein polyclonal antibody (1:50; Genscript), washed again, then incubated for 30 minutes with an anti-rabbit IgG-FITC conjugated secondary antibody (Invitrogen). The slides were washed in PBS, then counterstained with Hoechst, washed again, and then mounted with Gelvatol. Dried slides were imaged at 40x magnification using a Leica DM18 inverted fluorescence microscope and denoised using the Leica Application Suite X software.

### Real-time reverse transcription quantitative PCR (qPCR)

For qPCR experiments, cells were grown in 6 well plates (Neurons: 9.6 x 10^5^ cells/well. U2OS: 2 x 10^5^ cells/well). Cellular RNA was collected in Trizol (Life Technologies 15596018). Non-neuronal RNA was purified using a RNA clean & concentrator-25 kit (Zymo research R1018). Neuron RNA was purified by chloroform precipitation with glycogen addition. 1μg of RNA was used to synthesize complimentary DNA (cDNA) using Moloney Murine Leukemia Virus-Reverse Transcriptase (ThermoFisher scientific 28025013) and hexameric random primers (ThermoFisher scientific 48190011). cDNA was then diluted 1:5, and 5μL of diluted cDNA were loaded into 384 well qPCR plates in triplicate. Forward and reverse primers were diluted to 0.2μM in PowerSYBR Green master mix (Thermofisher scientific 4368577), and 5uL of the combined mix was added per well. A Thermofisher QuantStudio 6 RT-qPCR instrument was used to quantify cDNA amplification. Gene expression was normalized to GAPDH and relative fold change in expression was calculated using the ddCt method. Primer sequences are included in S1 Table.

### Plaque assay

Neurons or U2OS were treated and infected as indicated. Two hours after infection, inoculum was removed and replaced with fresh media (U2OS), or conditioned media from uninfected neurons. Drugs were not replenished. Supernatants were collected at the indicated time points and stored at -80°C. Infected supernatants or tissue homogenates were serially diluted in serum free media and 200uL was plated onto BHK cells (RVFV MP-12, LACV, CEV, PTV, VSV-RVFV) or Vero cells (RVFV ZH501). Virus was adsorbed for 45 minutes with rocking every 15 minutes. Virus inoculum was removed and cells were overlaid with MEM plus 5% FBS, 1% GlutaMAX, 1% Non-essential amino acids (Invitrogen 111140–050), and 0.65% agarose. 2–3 days later, cells were fixed with 2mL of 8% formaldehyde and plaques were visualized with 0.1% crystal violet staining.

### RNAseq

Neurons (9.6 x 10^5^ cells in duplicate wells) were treated with vehicle, 10μg/mL of Pam_3_CSK_4_ or Pam_2_CSK_4_, for 6h. RNA was collected in Trizol, and purified using a Zymo RNA clean & concentrator-25 kit, with duplicate wells pooled during purification. cDNA libraries were created with a TruSeq RNA library prep kit (Illumina) and sequenced on an Illumina NextSeq 500. Reads were aligned to the Rnor_6.0 genome with Kallisto [83], normalized with EdgeR [84], and differentially expressed genes were identified with Limma [85] using a linear model fit. Differentially expressed genes were used for pathway analysis, using Metascape [55].

### Viral neutralization assay

Pam_3_CSK_4_ was added to RVFV at 10μg/mL, and was incubated at 37°C for 4h. At the time of infection, the mixture was diluted to infect neurons at 0.3 MOI. This diluted the virus and ligand by more than 1000x. 24hpi, cells were fixed and infection was quantified by microscopy. As a positive control, a RVFV-neutralizing antibody (4D4) was added at 1:1000.

### Viral entry assays

Drugs at the indicated concentrations were added to cells in 6 well plates, and incubated for 1hr. Plates were sealed in plastic bags and cooled to 15°C for 30min. Virus was added at the indicated MOI, and was bound for 1hr for U2OS or 2h for neurons at 15°C, where viral entry does not occur. For viral attachment assays, virus was then aspirated from the cells and they were washed 2x with cold DPBS. Total RNA was then collected, and quantified by RT-qPCR. For viral uptake assays, cells were treated and virus was bound as above. Cells were then transferred to a 37°C incubator for 2hr to allow for viral entry. Non-internalized virus was removed by trypsinization (0.25% for 3 minutes at 37°C), and cells were washed before RNA was collected. Viral RNA was measured by qPCR. For acid bypass assays, 1 x 10^5^ U2OS cells in 12 well plates were pretreated with indicated drugs for 2 hours, before the addition of Bafilomycin A1 for 1hr. RVFV was bound to cells as above. Cells were washed 2x with DPBS, and then treated for 10 minutes with indicated treatments diluted in OptiMEM, pH adjusted to 7.6 or 5.2. Cells were washed, and complete media containing BafA or vehicle, and Pam_3_CSK_4_ or vehicle was replaced for a 24h incubation. Infection was measured by qPCR. For RVFV-DiD assays, cells were plated on coverslips and treated and cooled to 15 degrees C. RVFV-DiD was bound in the presence of vehicle, Pam_3_CSK_4_, or bafilomycin A1 for 1hr at 15°C. At this time point cells were either washed and fixed, or incubated at 37°C for the indicated times. Fixed cells were washed 3x with PBS without detergent, and DNA was stained with Hoechst 33342. Coverslips were then imaged by confocal microscopy.

### Animal studies

Female C57BL/6 (3–6 weeks old) were purchased from Jackson Laboratories and housed at the RBL, up to 5 to a cage in temperature-controlled rooms with a 12 hour day/ 12 hour night light schedule. To account for age-dependent effects, treatment groups of mice of similar age distributions were used for each experiment. Food (IsoPro Rodent 3000) and water were provided *ad libitum*. For Pam_3_CSK_4_ therapeutic studies, mice were intracranially injected at the intersection of the coronal and sagittal sutures of the skull with 10 μL, containing 1 or 5 PFU of RVFV ZH501 and 100 μg of Pam_3_CSK_4_. For untreated controls, RVFV was combined with PBS. Virus was diluted in D2 medium (DMEM, 2% (v/v) FBS, 1% L-glutamine, and 1% penicillin-streptomycin) and media without virus was delivered to uninfected animals. Mice were weighed daily and closely monitored for development of clinical signs of disease. Endpoint criteria, which prompt immediate euthanasia, were defined based on weight, appearance, ataxia, and anemia scores. Unless mice met the euthanasia criteria explained above, they were euthanized on 3dpi or on 10 dpi. Mice were anesthetized by inhalation of vaporized isoflurane (IsoThesia, Henry Schein). For all experiments, necropsy was performed upon euthanasia to collect brain, liver, and serum samples from the mice. For tissues, half of the sample was immediately frozen at -80°C for virological analysis by qRT-PCR and plaque assay, while the other half was fixed in fresh 4% paraformaldehyde (Sigma) for histological analyses. Liquid samples were immediately stored at -80°C prior to downstream virological analyses.

### Data analysis

Automated microscopy, plaque assay, and qPCR data were compiled and analyzed using Graphpad Prism 9. All statistical tests performed were two sided, and all replicate experiments represent independent biological replicates. RNAseq data were processed using RStudio 2021.09.02 and R 4.1.2.

## Supporting information

S1 Fig Neurons are not protected from RVFV infection by type I or II IFNs. **(A, B)** Immunofluorescence confocal microscopy images demonstrating untreated, RVFV-infected rat cortical neurons (A, MOI = 0.3), and human osteosarcoma U2OS cells (B, MOI = 1) at 24hpi. 94.5x magnification, scale bars = 20μm. MAP2 or phalloidin are stained magenta, as labelled, and RVFV Gn was stained in green. **(C)** Relative RVFV RNA at 24hpi in neurons pretreated with vehicle, or 12,000 U/mL of the indicated IFN for 4h before infection (MOI = 0.1). Bars = mean, error bars = SEM. ns = not significant, One-way ANOVA with Tukey’s multiple comparisons test.
(TIF)

S2 Fig RVFV screen in primary neurons, and validation of LPS-*Rs*. **(A, B)** Relative survival of neurons (A) or U2OS (B) treated with innate ligands and infected with RVFV as in Fig 2A–2C. The number of MAP2 positive nuclei (neurons) or nuclei (U2OS) were quantified by automated microscopy and automated cell scoring in four images per well, averaged, and set relative to vehicle treated, infected wells. Ligands that were cytotoxic in both replicates are marked. **(C)** Relative RVFV infection of neurons treated with LPS-*Rs* or LPS-*Rs* ultrapure at the indicated concentrations for 4h before infection (MOI 0.3). At 24hpi, infected neurons were quantified by automated microscopy and analysis, and set relative to vehicle treated infection. n = 4. **(D)** Survival relative to mock treated, infected cells from experiments described in Fig 3B. Rat neurons shown in red, mouse neurons in blue, U2OS in black. n = 3 (neurons) or 4 (U2OS). **(E)** Relative infection (red) and survival (black) of rat neurons treated with Pam_3_CSK_4_ before infection with VSV-GFP (MOI 0.2, 14hpi). Neurons were identified as MAP2 positive cells, and infection was marked by GFP expression. Fold change is calculated relative to vehicle (water). n = 4. **(F-J)** Quantification of viral RNA in neurons or U2OS treated with vehicle or Pam_3_CSK_4_ at 10 μg/mL 4h before infection. Infections were performed as in Fig 3J–3N. F: *P = .0166; G: ***P = .0067; H: ***P = .0071; I: ns *P* = .1307; J: *P = .0222. Symbols = mean, error bars = SEM. Statistical analyses were performed by Welch’s t test (F-J).
(TIF)

S3 Fig Structures of lipopeptides used in this study. Regions that diverge from racemic Pam_3_CSK_4_ are outlined in yellow.
(TIF)

S4 Fig NF-κB signaling is not required for antiviral activity. **(A, B)** Relative expression of *Tnfa* (A) or *Cxcl1* (B) RNA in rat neurons stimulated with 10μg/mL Pam_3_CSK_4_. RNA was collected at the indicated time post-stimulation, and RNA levels were quantified relative to *Gapdh*. A: vehicle vs. 4h ***P = .0004; vs 6h ***P = .0008; vs. 8h **P = .0012; vs. 12h **P = .0065; vs. 24h ns *P* = .0929. B: ****P< .0001. **(C)** Metascape pathway analysis showing enrichment of GO terms in genes induced after lipopeptide stimulation. Neurons were treated and RNAseq was performed as described in Fig 4A. DEGs were used to determine GO term clusters enriched by one or both lipopeptides. Color indicates significance of enrichment, gray = not enriched. **(D)** Relative expression of *Ifit2* RNA in rat neurons stimulated with 10μg/mL Pam_3_CSK_4_ or 12,000 U/mL universal IFNα. RNA was collected at the indicated time post-stimulation, and RNA levels were quantified relative to *Gapdh*. ****P< .0001. **(E-G)** Relative expression of IFIT1 (E), CXCL10 (F) or TNFa (G) RNA in U2OS stimulated with 10 μg/mL Pam_3_CSK_4_ or 12,000 U/mL uIFNα. RNA was collected at the indicated time post-stimulation, and RNA levels were quantified relative to GAPDH. E: *P = .0464; ****P< .0001. F: *P = .0233; ****P< .0001. G: **P = .0099; ***P = .0007. **(H,I)** Relative expression of *Tnfa* (H) *or Cxcl1* (I) in rat neurons treated with IKK 16 (3 μM) or TPCA-1 (6 μM) for 1hr prior to 4h Pam_3_CSK_4_ stimulation (10 μg/mL). Data collected and displayed as in (A-B). H: DMSO *P = .0192; TPCA ns *P*> .9999; IKK 16 ns *P* = .5124. I: DMSO vehicle vs. Pam_3_CSK_4_ **** *P*< .0001; TPCA vehicle vs. Pam_3_CSK_4_ ***P = .0007; IKK 16 vehicle vs. Pam_3_CSK_4_ **** *P*< .0001; DSMO Pam_3_CSK_4_ vs. TPCA Pam_3_CSK_4_ **P = .0026; DSMO Pam_3_CSK_4_ vs. IKK 16 Pam_3_CSK_4_ **P = .0036 **(J)** Neurons were treated and stimulated as in H, and then infected with RVFV (MOI 0.1) for 24h. Viral N RNA was quantified by qPCR, relative to *Gapdh*. DMSO **P = .0019; TPCA **P = .0056; IKK 16 **P = .0059. For statistical analyses, the following tests were used: One-way ANOVA with Tukey’s multiple comparisons test (A,D), two-way ANOVA with Dunnett’s multiple comparisons test (E-G), or two-way ANOVA with Šídák’s multiple comparisons test (H-J).
(TIF)

S5 Fig VSV-RVFV is sensitive to Pam_3_CSK_4_ in U2OS and TLR2 KO mouse neurons. **(A,B)** Relative VSV N RNA expression in U2OS cells treated with 10 μg/mL of Pam_3_CSK_4_ or Pam_2_CSK_4_ for 4h before infection with VSV (A, MOI = 0.6) or VSV-RVFV (B, MOI = 3) for 14h. Viral RNA levels were determined by qPCR, relative to GAPDH. A: Vehicle vs Pam_3_CSK_4_ ns *P* = .6817; Vehicle vs Pam_2_CSK_4_ ns *P* = .7503. B: Vehicle vs Pam_3_CSK_4_ **P = .002; Vehicle vs Pam_2_CSK_4_ *P = .028. **(C,D)** Relative levels of RVFV (C) or VSV N RNA (D) in wild type or TLR2 KO mouse cortical neurons, treated with 10 μg/mL Pam_3_CSK_4_ for 4h before infection with RVFV (24hpi, MOI 0.1) or VSV-RVFV (14hpi, MOI 7.5). Viral RNA was quantified relative to *28s* RNA. C: ns *P* = .0807, ** *P* = .0071. D: ***P = .0002, ****P< .0001. **(E)** VSV-RVFV titers from wild type or TLR2 KO mouse neurons treated with vehicle or 10 μg/mL Pam_3_CSK_4_ 4h before infection (MOI 7.5). Supernatants were collected at 15hpi. Titers were log_10_ transformed for statistical analysis. WT: **P = .0043; KO: **P = .0011. **(F)** Quantification of automated microscopy to detect VSV-RVFV infection (MOI 7.5) in WT or TLR2 KO mouse neurons. Cells were treated with indicated doses of Pam_3_CSK_4_ 4h before infection, and at 14hpi, automated microscopy and analysis were used to determine the percentage of infected neurons, relative to vehicle treated cells. n = 2. Symbols and bars = mean, error bars = SEM. Statistical analyses performed by one-way ANOVA with Tukey’s multiple comparisons test (A-B) or two-way ANOVA with Šídák’s multiple comparisons test (C-E).
(TIF)

S6 Fig Pam_3_CSK_4_ does not decrease RVFV binding or uptake. **(A)** Quantification of RVFV bound to rat neurons, by qPCR. Cells were treated as in Fig 5D, except that for the trypsin condition, cells were incubated with 0.05% trypsin for 3 minutes after viral binding but before RNA collection. Vehicle vs. Pam_3_CSK_4_: ns *P* = .363; vs. trypsin: ns *P* = .0795. **(B)** Quantification of RVFV uptake in rat neurons, by qPCR. Cells were treated as in Fig 5E, except for the no trypsin condition, in which cells were not treated with trypsin before RNA was collected. Vehicle vs. Pam_3_CSK_4_: ns *P* = .4773; vs. Pam_2_CSK_4_: ns *P* = .992; vs. no trypsin: ns *P* = .0563. **(C)** Quantification of RVFV bound to U2OS, by qPCR. Cells were treated and data analyzed as in (A). *P = .0285, **P = .0096. **(D)** Quantification of RVFV uptake in U2OS, by qPCR. Cells treated as in (B). ns *P* = .2915. Bars = mean, error bars = SEM. Statistical analyses performed with one-way ANOVA with Tukey’s multiple comparisons test (A-C), or Welch’s t test (D).
(TIF)

S7 Fig RVFV-DiD fusion is reduced in neurons by Pam_3_CSK_4_. **(A)** Confocal microscopy showing RVFV-DiD puncta in rat neurons. Nuclei are stained blue and DiD signal is shown in white. 60x magnification, scale bar = 50μm. Z-stacks were acquired and are shown as maximum projections. Images representative of five sites per condition, n = 3. **(B-D)** MetaXpress was used for quantification of 1h timepoint from (A), showing **(B)** the number of DiD puncta per cell, **(C)** the total area of DiD per cell, (**D**) the average intensity of DiD puncta within each cell. Each dot represents one cell. Vehicle: 473 cells analyzed, BafA: 348 cells analyzed, Pam_3_CSK_4_: 390 cells analyzed. For (C and D), cells with 0 puncta are not plotted due to the log Y axis but are included in analysis. Dotted lines represent median of 3 compiled experiments. One-way ANOVA with Dunnett’s multiple comparisons test. ****P <0.001.
(TIF)

S8 Fig Pam_3_CSK_4_ reduced RVFV infection and pathology *in vivo*. **(A)** Mice were infected with RVFV with vehicle or Pam_3_CSK_4_ as in (Fig 6A). At 3 dpi, RVFV titers in the liver were determined by plaque assay. Statistical analysis was performed by two-way ANOVA with Tukey’s multiple comparisons test. *P = .0120 **(B)** RVFV-induced infection and pathology in the brain and liver was observed by hematoxylin and eosin stain (20x) and **(C)** immunofluorescence microscopy (40x) of animals euthanized at 3 dpi. Prominent hemorrhages in the liver are marked by arrowheads. For immunofluorescence, sections were probed with a custom rabbit anti-RVFV nucleoprotein polyclonal antibody and then a FITC-conjugated secondary antibody (green) and nuclei were labelled blue with Hoechst.
(TIF)

S1 Table RT-qPCR primers used in this study. (PDF)

S1 Data Raw data generated in this study. (XLSX)
