# Supplementary figures and images for: The lipopeptide Pam3CSK4 inhibits Rift Valley fever virus infection and protects from encephalitis
Source: PLoS Pathog. 2024 Jun 27;20(6):e1012343. doi: 10.1371/journal.ppat.1012343 (PMC11236204; doi:10.1371/journal.ppat.1012343)

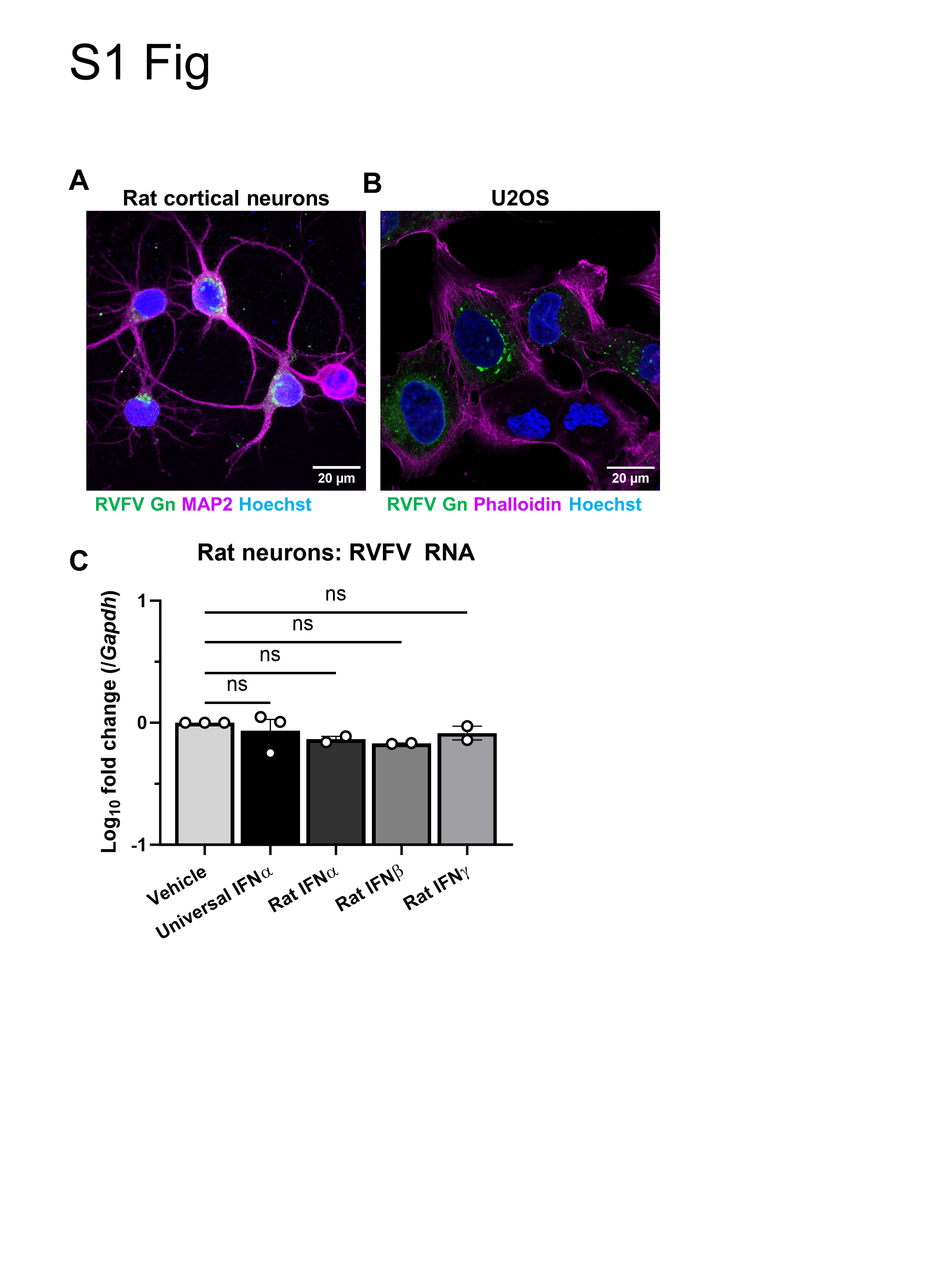

Supplement: S1 Fig — (A, B) Immunofluorescence confocal microscopy images demonstrating untreated, RVFV-infected rat cortical neurons (A, MOI = 0.3), and human osteosarcoma U2OS cells (B, MOI = 1) at 24hpi. 94.5x magnification, scale bars = 20μm. MAP2 or phalloidin are stained magenta, as labelled, and RVFV Gn was stained in green. (C) Relative RVFV RNA at 24hpi in neurons pretreated with vehicle, or 12,000 U/mL of the indicated IFN for 4h before infection (MOI = 0.1). Bars = mean, error bars = SEM. ns = not significant, One-way ANOVA with Tukey’s multiple comparisons test. (TIF) [file ppat.1012343.s001.tif]

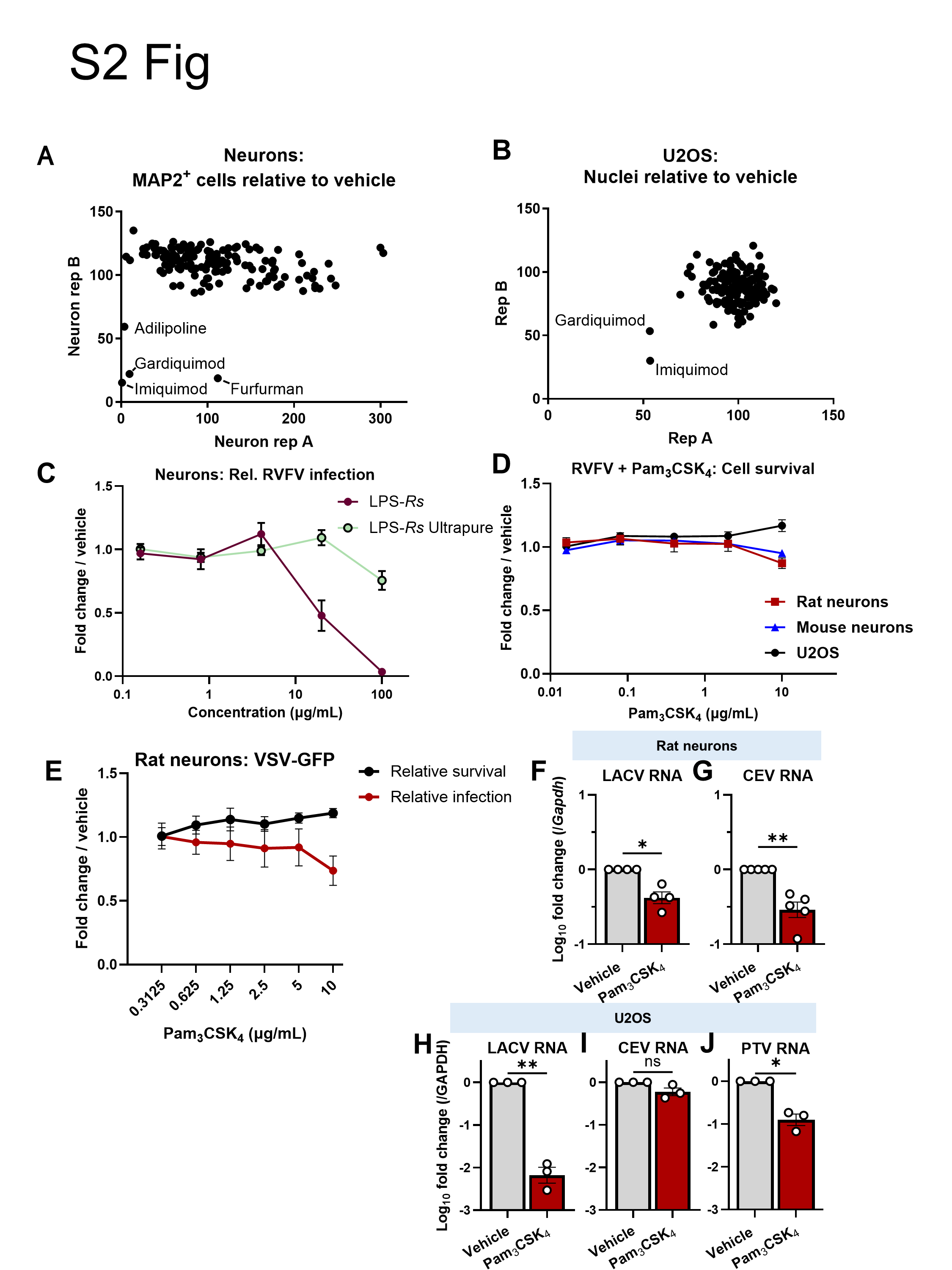

Supplement: S2 Fig — (A, B) Relative survival of neurons (A) or U2OS (B) treated with innate ligands and infected with RVFV as in Fig 2A–2C. The number of MAP2 positive nuclei (neurons) or nuclei (U2OS) were quantified by automated microscopy and automated cell scoring in four images per well, averaged, and set relative to vehicle treated, infected wells. Ligands that were cytotoxic in both replicates are marked. (C) Relative RVFV infection of neurons treated with LPS-Rs or LPS-Rs ultrapure at the indicated concentrations for 4h before infection (MOI 0.3). At 24hpi, infected neurons were quantified by automated microscopy and analysis, and set relative to vehicle treated infection. n = 4. (D) Survival relative to mock treated, infected cells from experiments described in Fig 3B. Rat neurons shown in red, mouse neurons in blue, U2OS in black. n = 3 (neurons) or 4 (U2OS). (E) Relative infection (red) and survival (black) of rat neurons treated with Pam3CSK4 before infection with VSV-GFP (MOI 0.2, 14hpi). Neurons were identified as MAP2 positive cells, and infection was marked by GFP expression. Fold change is calculated relative to vehicle (water). n = 4. (F-J) Quantification of viral RNA in neurons or U2OS treated with vehicle or Pam3CSK4 at 10 μg/mL 4h before infection. Infections were performed as in Fig 3J–3N. F: *P = .0166; G: ***P = .0067; H: ***P = .0071; I: ns P = .1307; J: *P = .0222. Symbols = mean, error bars = SEM. Statistical analyses were performed by Welch’s t test (F-J). (TIF) [file ppat.1012343.s002.tif]

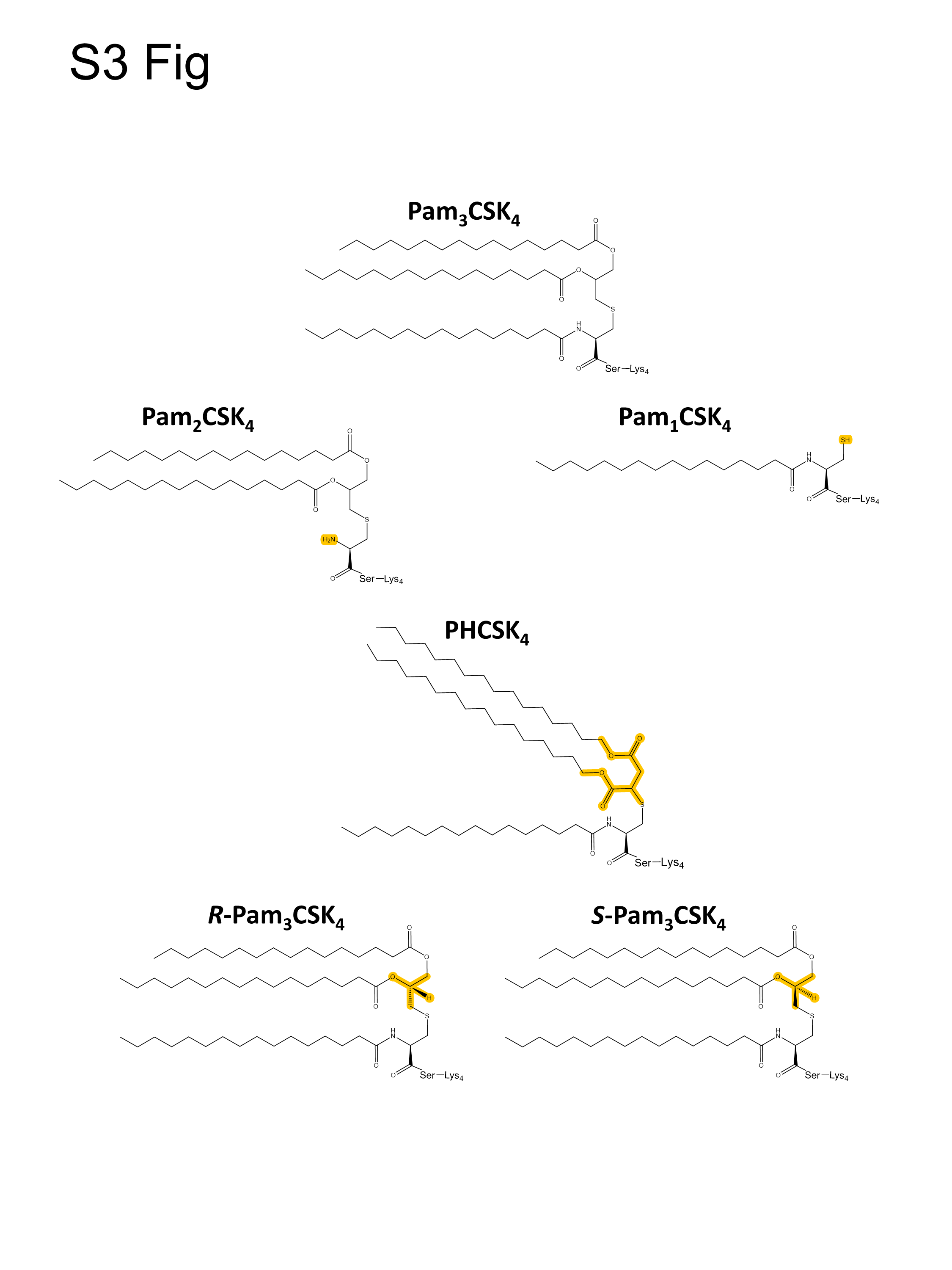

Supplement: S3 Fig — Regions that diverge from racemic Pam3CSK4 are outlined in yellow. (TIF) [file ppat.1012343.s003.tif]

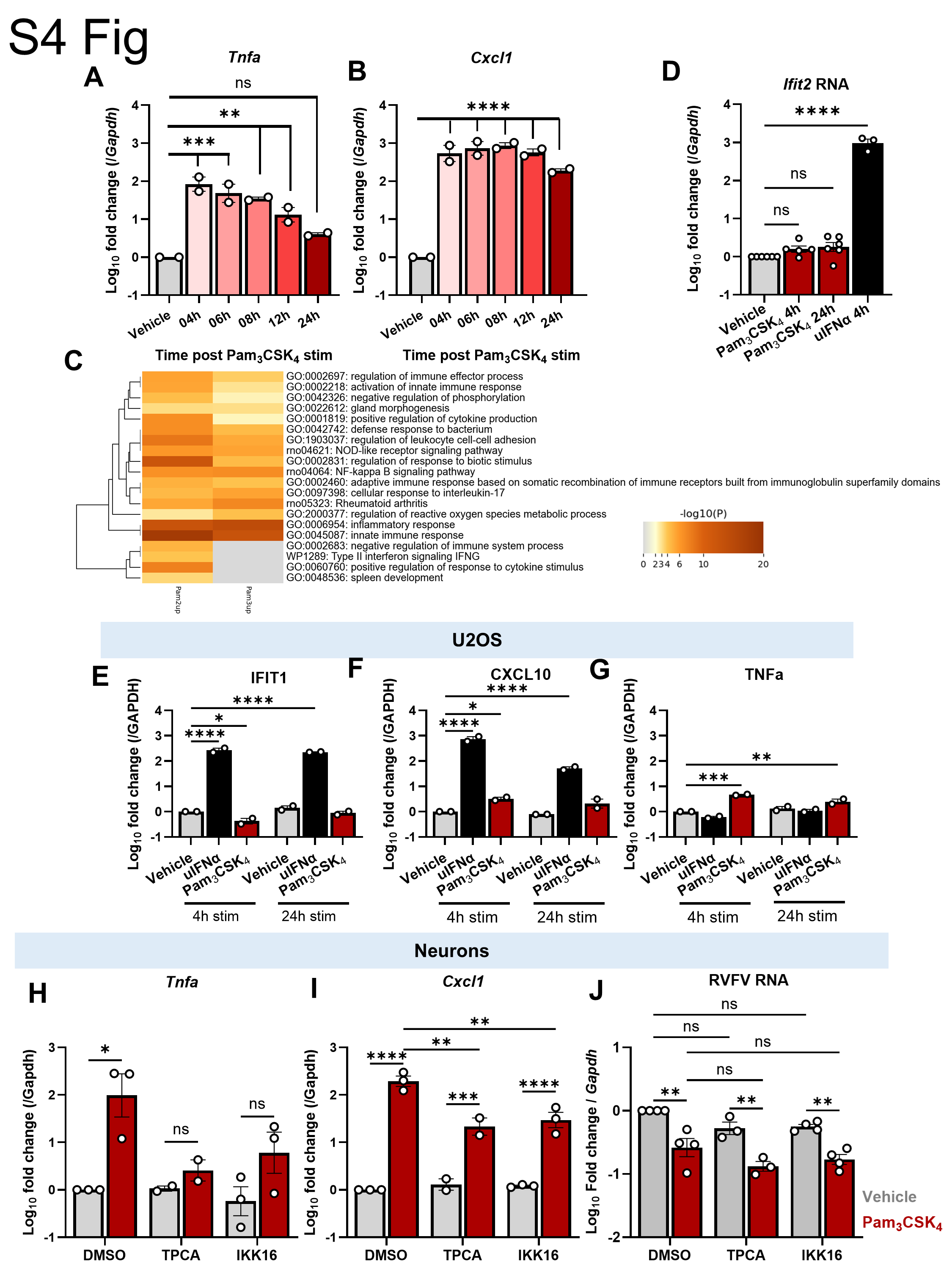

Supplement: S4 Fig — (A, B) Relative expression of Tnfa (A) or Cxcl1 (B) RNA in rat neurons stimulated with 10μg/mL Pam3CSK4. RNA was collected at the indicated time post-stimulation, and RNA levels were quantified relative to Gapdh. A: vehicle vs. 4h ***P = .0004; vs 6h ***P = .0008; vs. 8h **P = .0012; vs. 12h **P = .0065; vs. 24h ns P = .0929. B: ****P< .0001. (C) Metascape pathway analysis showing enrichment of GO terms in genes induced after lipopeptide stimulation. Neurons were treated and RNAseq was performed as described in Fig 4A. DEGs were used to determine GO term clusters enriched by one or both lipopeptides. Color indicates significance of enrichment, gray = not enriched. (D) Relative expression of Ifit2 RNA in rat neurons stimulated with 10μg/mL Pam3CSK4 or 12,000 U/mL universal IFNα. RNA was collected at the indicated time post-stimulation, and RNA levels were quantified relative to Gapdh. ****P< .0001. (E-G) Relative expression of IFIT1 (E), CXCL10 (F) or TNFa (G) RNA in U2OS stimulated with 10 μg/mL Pam3CSK4 or 12,000 U/mL uIFNα. RNA was collected at the indicated time post-stimulation, and RNA levels were quantified relative to GAPDH. E: *P = .0464; ****P< .0001. F: *P = .0233; ****P< .0001. G: **P = .0099; ***P = .0007. (H,I) Relative expression of Tnfa (H) or Cxcl1 (I) in rat neurons treated with IKK 16 (3 μM) or TPCA-1 (6 μM) for 1hr prior to 4h Pam3CSK4 stimulation (10 μg/mL). Data collected and displayed as in (A-B). H: DMSO *P = .0192; TPCA ns P> .9999; IKK 16 ns P = .5124. I: DMSO vehicle vs. Pam3CSK4 **** P< .0001; TPCA vehicle vs. Pam3CSK4 ***P = .0007; IKK 16 vehicle vs. Pam3CSK4 **** P< .0001; DSMO Pam3CSK4 vs. TPCA Pam3CSK4 **P = .0026; DSMO Pam3CSK4 vs. IKK 16 Pam3CSK4 **P = .0036 (J) Neurons were treated and stimulated as in H, and then infected with RVFV (MOI 0.1) for 24h. Viral N RNA was quantified by qPCR, relative to Gapdh. DMSO **P = .0019; TPCA **P = .0056; IKK 16 **P = .0059. For statistical analyses, the following tests were used: One-way ANOVA wi [file ppat.1012343.s004.tif]

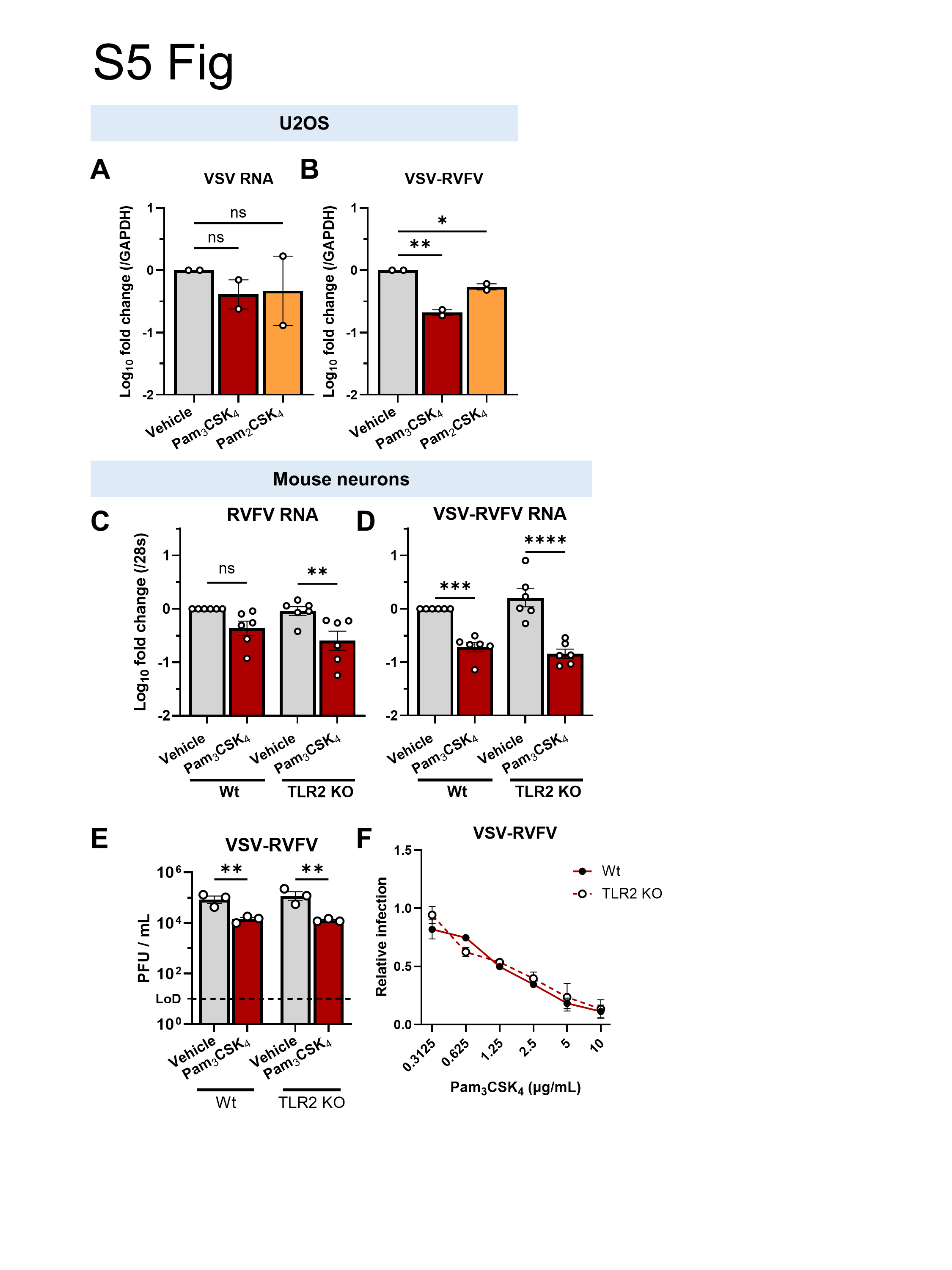

Supplement: S5 Fig — (A,B) Relative VSV N RNA expression in U2OS cells treated with 10 μg/mL of Pam3CSK4 or Pam2CSK4 for 4h before infection with VSV (A, MOI = 0.6) or VSV-RVFV (B, MOI = 3) for 14h. Viral RNA levels were determined by qPCR, relative to GAPDH. A: Vehicle vs Pam3CSK4 ns P = .6817; Vehicle vs Pam2CSK4 ns P = .7503. B: Vehicle vs Pam3CSK4 **P = .002; Vehicle vs Pam2CSK4 *P = .028. (C,D) Relative levels of RVFV (C) or VSV N RNA (D) in wild type or TLR2 KO mouse cortical neurons, treated with 10 μg/mL Pam3CSK4 for 4h before infection with RVFV (24hpi, MOI 0.1) or VSV-RVFV (14hpi, MOI 7.5). Viral RNA was quantified relative to 28s RNA. C: ns P = .0807, ** P = .0071. D: ***P = .0002, ****P< .0001. (E) VSV-RVFV titers from wild type or TLR2 KO mouse neurons treated with vehicle or 10 μg/mL Pam3CSK4 4h before infection (MOI 7.5). Supernatants were collected at 15hpi. Titers were log10 transformed for statistical analysis. WT: **P = .0043; KO: **P = .0011. (F) Quantification of automated microscopy to detect VSV-RVFV infection (MOI 7.5) in WT or TLR2 KO mouse neurons. Cells were treated with indicated doses of Pam3CSK4 4h before infection, and at 14hpi, automated microscopy and analysis were used to determine the percentage of infected neurons, relative to vehicle treated cells. n = 2. Symbols and bars = mean, error bars = SEM. Statistical analyses performed by one-way ANOVA with Tukey’s multiple comparisons test (A-B) or two-way ANOVA with Šídák’s multiple comparisons test (C-E). (TIF) [file ppat.1012343.s005.tif]

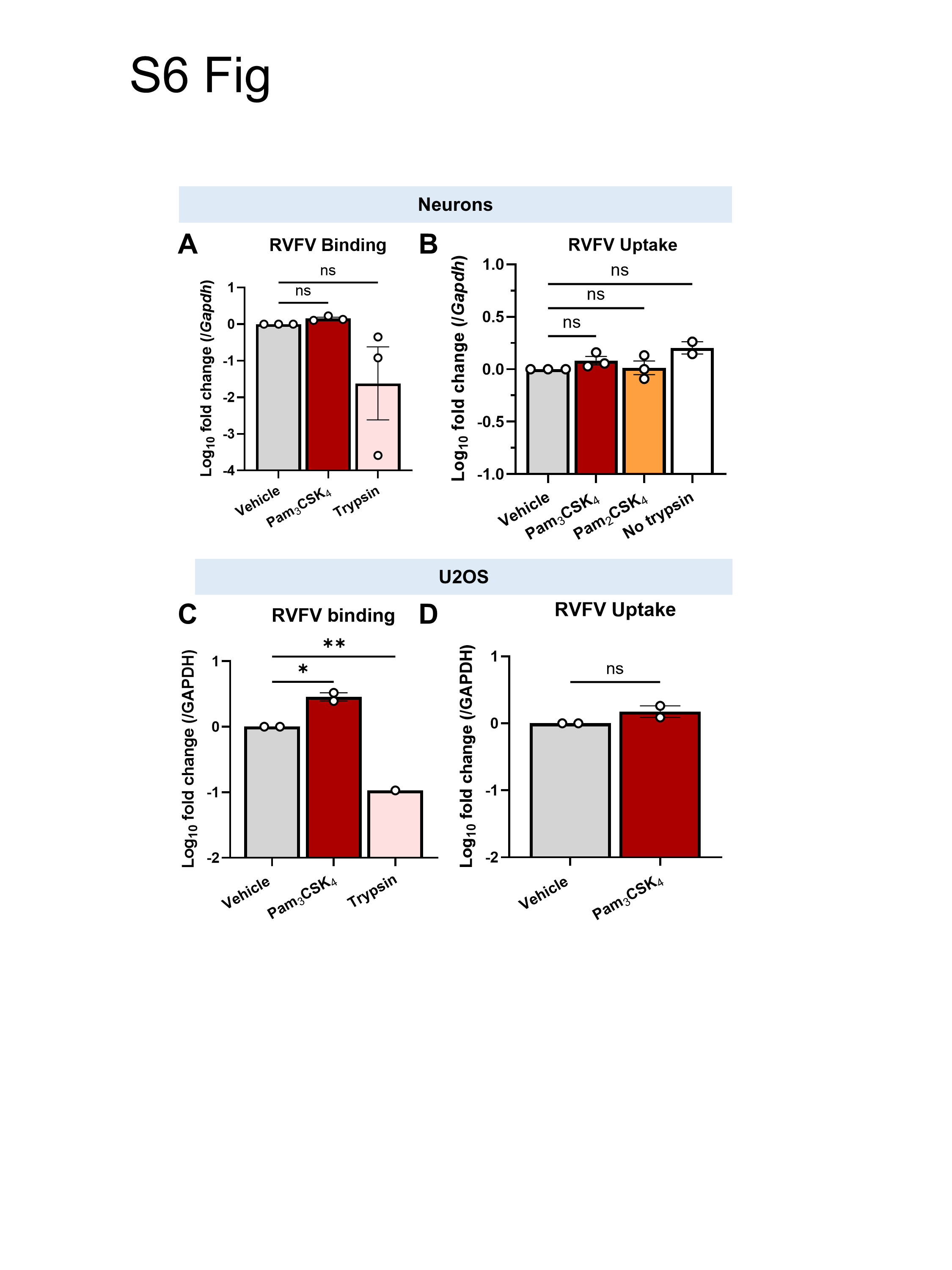

Supplement: S6 Fig — (A) Quantification of RVFV bound to rat neurons, by qPCR. Cells were treated as in Fig 5D, except that for the trypsin condition, cells were incubated with 0.05% trypsin for 3 minutes after viral binding but before RNA collection. Vehicle vs. Pam3CSK4: ns P = .363; vs. trypsin: ns P = .0795. (B) Quantification of RVFV uptake in rat neurons, by qPCR. Cells were treated as in Fig 5E, except for the no trypsin condition, in which cells were not treated with trypsin before RNA was collected. Vehicle vs. Pam3CSK4: ns P = .4773; vs. Pam2CSK4: ns P = .992; vs. no trypsin: ns P = .0563. (C) Quantification of RVFV bound to U2OS, by qPCR. Cells were treated and data analyzed as in (A). *P = .0285, **P = .0096. (D) Quantification of RVFV uptake in U2OS, by qPCR. Cells treated as in (B). ns P = .2915. Bars = mean, error bars = SEM. Statistical analyses performed with one-way ANOVA with Tukey’s multiple comparisons test (A-C), or Welch’s t test (D). (TIF) [file ppat.1012343.s006.tif]

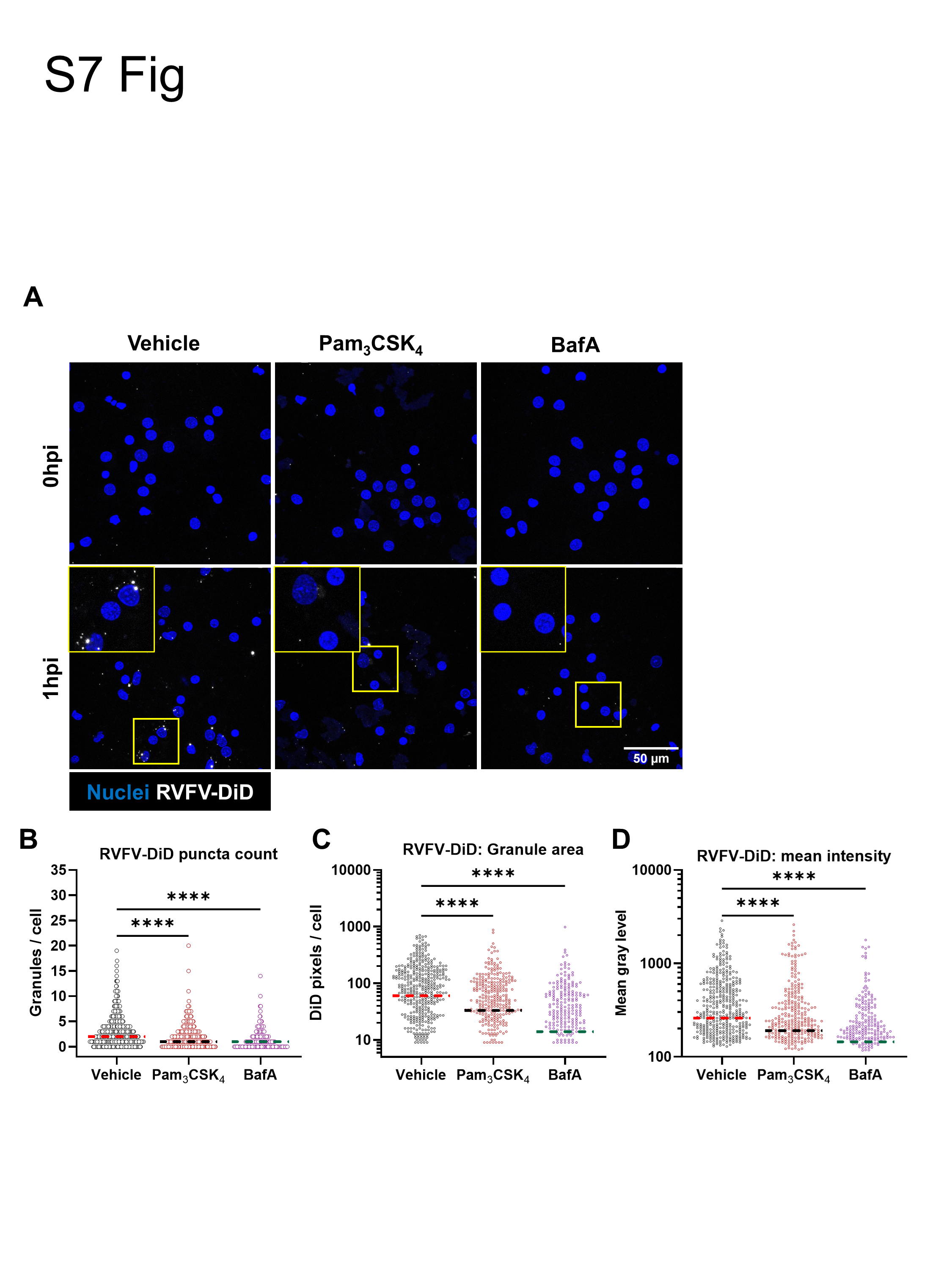

Supplement: S7 Fig — (A) Confocal microscopy showing RVFV-DiD puncta in rat neurons. Nuclei are stained blue and DiD signal is shown in white. 60x magnification, scale bar = 50μm. Z-stacks were acquired and are shown as maximum projections. Images representative of five sites per condition, n = 3. (B-D) MetaXpress was used for quantification of 1h timepoint from (A), showing (B) the number of DiD puncta per cell, (C) the total area of DiD per cell, (D) the average intensity of DiD puncta within each cell. Each dot represents one cell. Vehicle: 473 cells analyzed, BafA: 348 cells analyzed, Pam3CSK4: 390 cells analyzed. For (C and D), cells with 0 puncta are not plotted due to the log Y axis but are included in analysis. Dotted lines represent median of 3 compiled experiments. One-way ANOVA with Dunnett’s multiple comparisons test. ****P <0.001. (TIF) [file ppat.1012343.s007.tif]

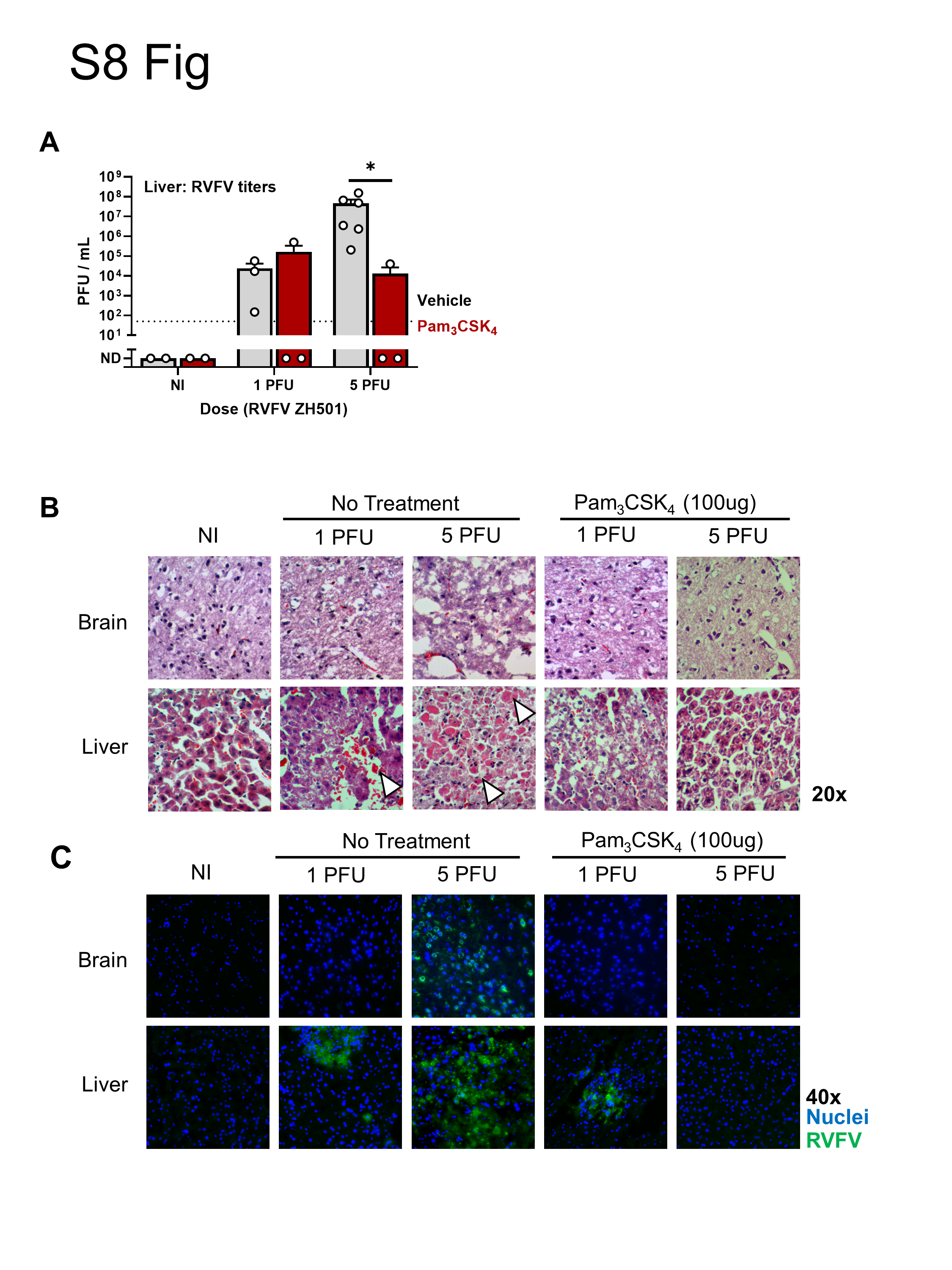

Supplement: S8 Fig — (A) Mice were infected with RVFV with vehicle or Pam3CSK4 as in (Fig 6A). At 3 dpi, RVFV titers in the liver were determined by plaque assay. Statistical analysis was performed by two-way ANOVA with Tukey’s multiple comparisons test. *P = .0120 (B) RVFV-induced infection and pathology in the brain and liver was observed by hematoxylin and eosin stain (20x) and (C) immunofluorescence microscopy (40x) of animals euthanized at 3 dpi. Prominent hemorrhages in the liver are marked by arrowheads. For immunofluorescence, sections were probed with a custom rabbit anti-RVFV nucleoprotein polyclonal antibody and then a FITC-conjugated secondary antibody (green) and nuclei were labelled blue with Hoechst. (TIF) [file ppat.1012343.s008.tif]
