# Supplementary material for: The lipopeptide Pam3CSK4 inhibits Rift Valley fever virus infection and protects from encephalitis
Source: PLoS Pathog. 2024 Jun 27;20(6):e1012343. doi: 10.1371/journal.ppat.1012343 (PMC11236204; doi:10.1371/journal.ppat.1012343)
Supplement: S1 Table — (PDF) [file ppat.1012343.s009.pdf]

Primer name	Species	Primer sequence (5'-3')
GAPDH_F	Homo sapiens	ACCAAATCCGTTGACTCCGACCTT
GAPDH_R	Homo sapiens	TCGACAGTCAGCCGCATCTTCTTT
IFIT1_F	Homo sapiens	CAACCAAGCAAATGTGAGGA
IFIT1_R	Homo sapiens	AGGGGAAGCAAAGAAAATGG
CXCL10_F	Homo sapiens	GTGGCATTCAAGGAGTACCTC
CXCL10_R	Homo sapiens	TGATGGCCTTCGATTCTGGATT
TNFa_F	Homo sapiens	CCTCTCTCTAATCAGCCCTCTG
TNFa_R	Homo sapiens	GAGGACCTGGGAGTAGATGAG
Gapdh_F	Rattus norvegicus	GACATGCCGCCTGGAGAAAC
Gapdh_R	Rattus norvegicus	AGCCCAGGATCGCCTTTACGT
Ifit2_F	Rattus norvegicus	CAGCAGTCATGAGTACAGCC
Ifit2_R	Rattus norvegicus	GTCTTCGGCTTCCCCTAAGC
Cxcl10_F	Rattus norvegicus	CTGAGTGGGACTCAAGGGAT
Cxcl10_R	Rattus norvegicus	TCTTTGGCTCACCGCTTTCA
Tnfa_F	Rattus norvegicus	CACGTCGTAGCAAACCACCAA
Tnfa_R	Rattus norvegicus	AAGGGCTCTTGATGGCAGAG
Cxcl1_F	Rattus norvegicus	CCAAACCGAAGTCATAGCCAC
Cxcl1_R	Rattus norvegicus	TGTCAGAAGCCAGCGTTCAC
28s_F	Mus musculus	GGTTGAGGGCCACCTTATTT
28s_R	Mus musculus	GAAGAAAGACCGGGAAGAGAAA
Cxcl1_F	Mus musculus	ACCCAAACCGAAGTCATAGCC
Cxcl1_R	Mus musculus	TTGTCAGAAGCCAGCGTTCA
Ccl2_F	Mus musculus	CACTCACCTGCTGCTACTCA
Ccl2_R	Mus musculus	GCTTGGTGACAAAAACTACAGC
RVFV N_F	RVFV MP-12	CAAGCAGTGGACCGCAATGAGA
RVFV N_F	RVFV MP-12	GGGCTTGTTGCCACGAGTTAGA
LACV N_F	LACV Original	GCTGAGTCTAATGGTGTAGGATG
LACV N_R	LACV Original	TGGTCAGCGGGTAGAATTTG
CEV N_F	CEV BFS-283	CCACCGCATCTCAGGATACC
CEV N_R	CEV BFS-283	TGACTCCGCAATTGGGTTGA
PTV N_F	PTV Balliet	CCAGCAGTTATCCTAGGGCG
PTV N_R	PTV Balliet	ATTGGTCGCTCAAAGCTGGA
VSV N_F	VSV Indiana	CGGAGGATTGACGACTAATGC
VSV N_R	VSV Indiana	ACCATCCGAGCCATTCGA
